# Functional screening of mammalian mechanosensitive genes using *Drosophila* RNAi library– *Smarcd3/Bap60* is a mechanosensitive pro-inflammatory gene

**DOI:** 10.1038/srep36461

**Published:** 2016-11-07

**Authors:** Sandeep Kumar, In-hwan Jang, Chan Woo Kim, Dong-Won Kang, Won Jae Lee, Hanjoong Jo

**Affiliations:** 1 Wallace H. Coulter Department of Biomedical Engineering, Georgia Institute of Technology and Emory University, Atlanta, GA, USA; 2 National Creative Research Initiative Center for Hologenomics, and School of Biological Sciences, Seoul National University, Seoul 151-742, South Korea; 3 Division of Cardiology, Department of Medicine, Emory University, Atlanta, GA, USA

## Abstract

Disturbed blood flow *(d-flow)* induces atherosclerosis by altering the expression of mechanosensitive genes in the arterial endothelium. Previously, we identified >580 mechanosensitive genes in the mouse arterial endothelium, but their role in endothelial inflammation is incompletely understood. From this set, we obtained 84 *Drosophila* RNAi lines that silences the target gene under the control of upstream activation sequence (UAS) promoter. These lines were crossed with C564-GAL4 flies expressing GFP under the control of *drosomycin* promoter, an NF-κB target gene and a marker of pathogen-induced inflammation. Silencing of *psmd12* or *ERN1* decreased infection-induced *drosomycin* expression, while *Bap60* silencing significantly increased the *drosomycin* expression. Interestingly, knockdown of Bap60 in adult flies using temperature-inducible Bap60 RNAi (*C564*^*ts*^*-GAL4-Bap60-RNAi*) enhanced *drosomycin* expression upon Gram-positive bacterial challenge but the basal *drosomycin* expression remained unchanged compared to the control. In the mammalian system, smarcd3 (mammalian ortholog of Bap60) expression was reduced in the human- and mouse aortic endothelial cells exposed to oscillatory shear *in vitro as well as in the d-flow* regions of mouse arterial endothelium *in vivo.* Moreover, siRNA-mediated knockdown of smarcd3 induced endothelial inflammation. In summary, we developed an *in vivo Drosophila* RNAi screening method to identify flow-sensitive genes that regulate endothelial inflammation.

Vascular endothelial cells respond to blood flow through mechanosensors, which transduce the mechanical force associated with flow (known as shear stress) into cell signaling events and ultimately to changes in gene expression^1^,^2^,^3^. Disturbed blood flow *(d-flow)*, which occurs in branched and curved arteries, is characterized by complex flow patterns with low magnitude and oscillatory shear stress (OS), whereas stable flow (*s-flow*) is characterized by high magnitude, unidirectional laminar shear stress (LS) due to blood flow in the straight sections of the vasculature. *D-flow* and *s-flow* promote and suppress endothelial inflammation and atherosclerosis, respectively^4^,^5^,^6^,^7^. The molecular mechanisms underlying the pro-inflammatory effects of *d-flow* on the endothelium are likely to be regulated by flow-sensitive genes, but the specific mechanisms are still unclear^6^,^8^.

Previously, we carried out a transcriptomic study to determine which genes were regulated by blood flow using a partial carotid ligation model of atherosclerosis^6^. From this study, we identified more than 580 mechanosensitive genes that changed in response to *d-flow* as compared to *s-flow*. However, it remains unclear which of these mechanosensitive genes regulate endothelial inflammation, a key proatherogenic response.

In *Drosophila*, genetic analyses reveal that there are two independent NF-κB signaling pathways, Toll-mediated and immune deficiency (IMD)-mediated pathways^9^,^10^. Toll pathway is activated by Gram-positive bacteria or fungi leading to the activation of Dif, a p65-like NF-κB, whereas IMD-pathway is activated by Gram-negative bacteria leading to the activation of Relish, a p105-like NF-κB^11^. NF-κB-dependent antimicrobial peptide (AMP) gene expression such as Dif-dependent *drosomycin* and Relish-dependent *diptericin* is shown to be required for efficient antimicrobial responses^12^.

To functionally screen candidate mechanosensitive genes that may regulate endothelial inflammation, we have developed an *in vivo* Toll-mediated NF-κB activation screening assay using *Drosophila* carrying a fluorescent NF-κB target gene reporter [*drosomycin* promoter fused to Green Fluorescent Protein (GFP)] along with RNAi targeting the respective mechanosensitive gene under the control of the *UAS-GAL4* promoter^13^,^14^,^15^.

Using this *in vivo* functional screening method, we screened 84 RNAi fly lines and identified novel, flow-sensitive, pro-inflammatory genes including *smarcd3* (mammalian ortholog of *Bap60*) that regulates Toll-mediated NF-κB activation.

## Materials and Methods

### Animal Study Design

Animals were maintained and cared for in accordance to the National Institutes of Health (NIH) guidelines in our AAALAC-accredited experimental animal facility under a controlled environment (21° ± 2 °C, 50% ± 10% relative humidity and a 12-h light:12-h dark cycle with lights on at 0700 h EST). The Institutional Animal Care and Use Committee (IACUC) at Emory University approved the project protocols and procedures performed in accordance with the established guidelines and regulations consistent with federal assurance. Male C57BL/6 and *ApoE*^***−/−***^ mice (Jackson Laboratory) were fed *ad libitum* with standard chow diet until surgery at 8–9 weeks of age. Mice were anaesthetized with 3.5% isoflurane initially and then 1.5–2% during the entire procedure and underwent partial ligation as we previously described^16^. Following surgery, analgesic buprenorphine (0.1 mg kg^−1^) was administered. Following the partial carotid ligation, *d-flow* in LCA was confirmed by Doppler ultrasonography (Vevo1100, Visualsonics)^16^,^17^. The detailed method for endothelial-enriched RNA extraction, and microarray processing and analyses have been previously described in detail in our previous publications^6^,^8^,^18^.

### Generation of *Drosophila* lines carrying RNAi and GFP-NF-κB reporter

We first determined which of the 588 flow-sensitive murine genes previously identified^6^ have corresponding fly orthologs by comparing amino acid homology between murine and *Drosophila* genes (http://flybase.org)^19^. Through this manual comparison, we identified 99 *Drosophila* genes having significant sequence homology to corresponding murine genes. Of these, we obtained 84 *Drosophila* RNAi lines expressing corresponding hairpin RNA (Supplementary Table 1). These double-stranded RNAs are processed by Dicer into siRNAs which direct sequence-specific degradation of the target mRNA (UAS-RNAi flies) from Vienna *Drosophila* Resource Center (VDRC).

To develop fly lines that can report onset of inflammation by NF-κB activation as a surrogate marker, we used *C564-GAL4* flies carrying *drosomycin-GFP* (GFP under control of *drosomycin* promoter) reporter. *Drosomycin* is a well-known NF-κB target gene in flies. This allowed us to test the effect of individual RNAi on the Toll-mediated NF-κB activation in the absence or presence of bacterial challenge. To this end, we mated male UAS-RNAi lines to female *C564-GAL4* flies expressing *drosomycin-GFP*. All fly lines were maintained at 25 °C with standard fly medium^20^.

For validation studies, conditional expression of UAS-RNAi was achieved using temperature-sensitive allele of GAL80 under the control of tubulin promoter (*tub-GAL80*^*ts*^). In these *C564-GAL4-Tub-GAL80*^*ts*^ *(C564ts-GAL4)* flies, GAL80ts (inhibitor of GAL4 activity) is active at 18 °C and thus capable of inhibiting GAL4 activity^21^. At 29 °C, the GAL80ts becomes inactive allowing RNAi expression. Flies were grown at 18 °C during development and adult flies were shifted to 29 °C for 5 days for the expression of UAS-RNAi before infection experiment.

### *In vivo* GFP screening by fluorescence stereo microscopy

GFP expression in the flies was examined under basal and bacterial infected conditions by fluorescence stereo microscopy. A total of three or more independent cohorts of ~10 adult flies each were examined before and 24 h post-bacterial infection. A representative GFP fluorescence screening set was blindly scored on a scale of 0 (least) to 5 (brightest) by 4 observers for semi-quantitation of fluorescence intensity.

### Infection Experiments

Bacterial infection was performed by pricking adult flies in the thorax with a thin needle previously dipped in a concentrated pellet of a Gram-positive bacteria (*Micrococcus luteus*) culture (OD = 200) or Gram-negative bacteria (*Erwinia carotovora 15*) as previously described^20^. Each experiment was performed with approximately 10 flies for each genotype, and the results shown are representative of at least three independent tests. The lethal RNAi lines are shown in Supplementary Table 1 and were not pursued further.

### Cell culture and *in vitro* shear stress system

Human umbilical vein endothelial cells (HUVECs), human aortic endothelial cells (HAECs) and immortalized mouse aortic endothelial cells (iMAECs) were maintained in their respective culture medium as we previously described^22^,^23^,^24^,^25^. Shear stress was applied to the cells in 10-cm culture plates for 24 hours using a cone-and-plate viscometer that exerts 15 dyn/cm^2^ of unidirectional flow (laminar shear or LS) and ±5 dyn/cm^2^ of oscillatory shear stress (OS), respectively, as we previously described^26^,^27^. The cell culture plates were secured in place at the bottom by a vacuum pump, and the incubator was maintained at 5% CO2 and 37 °C. Cells with no shear stress (Static) were used as controls. Total RNA was collected by scraping the cells in QIAzol buffer (Qiagen).

### Quantitative real-time PCR (qPCR) assays

qPCR was performed to validate the functional screening results in *Drosophila*. The transcript level of *drosomycin* was measured in the RNA extracted from RNAi flies in either basal, 6hr or 24hr post-bacterial infection. qPCR assays were also performed using total RNAs obtained from the mouse carotid arterial endothelium and from endothelial cells exposed to shear stress *in vitro*. The primers used for qPCR analyses are listed in Supplementary Tables 2 and 3.

### Immunofluorescence staining

For *en face* confocal microscopy imaging, the right (RCA) and left carotid arteries (LCA) obtained at 48 hours post-ligation from C57Bl6 mice subjected to partial carotid ligation were used. The carotids were opened longitudinally and fixed on black wax with the endothelial layer facing upwards. The tissues were permeabilized with 0.25% (v/v) Triton X-100 in PBS, washed, blocked for 1.5 h in 10% (v/v) goat serum, and incubated with Rabbit polyclonal anti-smarcd3 antibody (Abcam; ab115917, dilution 1:100) in blocking solution (overnight, 4 °C). After additional washing, the tissues were incubated with DyLight 550-conjugated donkey anti-Rabbit secondary antibody (Abcam; dilution 1:250) in PBS for 2 h at room temperature, and with 4′,6-diamidino-2-phenylindole (DAPI) (Sigma-Aldrich) in PBS for 20 min. Fluorescence was imaged using a confocal microscope (Carl Zeiss; LSM 510 Meta instrument). Negative control tissues were stained with appropriate IgG isotype control antibodies (Santa Cruz Biotechnology). Nuclei were stained with DAPI.

### Monocyte adhesion assay

Human peripheral blood mononuclear leukocytes (THP-1 cells) were cultured in serum-containing RPMI medium (MT10040CM; Fisher) and were labeled in serum-free RPMI with 5 μl/ml 2′7′-bis-(2-carboxyethyl)-5-(and-6)-carboxyfluorescein (BCECF-AM, B-1170; Molecular Probes) at 37 °C for 30 minutes. HUVECs were transfected with either 150 nM *smarcd3-*siRNA (Dharmacon RNAi ON-TARGET plus SMART pool) or control siRNA for 24 hours. As a positive control, HUVECs were activated with 3 ng/ml TNFα in serum-free RPMI for 3 hours to induce monocyte adhesion. 5 × 10^5^ THP-1 cells per milliliter (in a total of 6 ml) were incubated with the HUVECs for 30 minutes at 37 °C. Nonadherent monocytes were washed away with HBSS (Corning), and the bound monocytes were fixed with 4% paraformaldehyde for 5 minutes. Bound monocytes were imaged in 8–10 fields per plate using fluorescent and bright-field microscopy, and quantification was done by ImageJ software (NIH)^28^.

### *Ex vivo* Aorta tissue culture and siRNA transfection

After sacrificing C57BL/6 mice, isolated aortas were longitudinally halved, opened *en-face* and cultured in a 48-well plate with 150 μl per well of complete medium viz. Dulbecco’s Modified Eagle’s Medium (DMEM, GIBCO, NY) containing 10% (*v/v*) fetal bovine serum, penicillin/streptomycin, and L-glutamine^29^. Transient transfections were performed using siRNA for 100 nM *smarcd3* (5′- GAGUACAUCAAUGGCGACAAGUAU-3′) or control for 48 h using Lipofectamine RNAiMAX (Invitrogen), following manufacturer’s protocol as described previously^8^. Post transfection, the *en face* immunostaining was performed for studying the expression of smarcd3 and vascular cell adhesion molecule (VCAM1). Signal intensities indicating the expression of VCAM1 (Cyan) and Smarcd3 (Red) were quantified by ImageJ software (NIH)^28^.

### *Ex vivo* endothelium monocyte adhesion assay

The abdominal aortas were isolated from C57BL/6 mice and the surrounding adventitial tissue was gently cleaned. siRNA for *smarcd3* or non-targeting controls were transfected as described above for 48 hours. The aortas were opened longitudinally and fixed *en face* with the endothelial surface-side up using fine needles on a black wax petri dish. A mouse monocyte macrophage cell line J774A.1 (ATCC^®^ TIB-67™) was labeled with Calcein-AM (Molecular probes), and labeled cells (5 × 10^5^) were incubated on the excised aortas that was pinned on the black wax dish for 30 min at 37 °C in a CO_2_ incubator^30^. These were then washed twice with phosphate buffered saline (PBS) and the bound J774A.1 cells were counted as above.

### Statistical analyses

Statistical analyses were carried out with Graph-Pad Prism (GraphPad Software). All error bars reported are standard error of mean (S.E.M) unless otherwise indicated. Pairwise comparisons were performed using one-way Student’s *t*-tests. Differences between groups were considered significant at P-values below 0.05.

## Results

### Selection of *Drosophila* RNAi lines for functional screening

Previously, we developed a mouse model of *d-flow*-induced atherosclerosis by the partial carotid ligation surgery^6^,^16^,^17^. Using this mouse model, we identified 588 mechanosensitive genes in the mouse arterial endothelium^6^. Although it is well-known that *d-flow* induces endothelial inflammation including NF-κB activation, the role of these mechanosensitive genes is largely unclear. To functionally identify mechanosensitive genes that are involved in NF-κB-dependent inflammation, we developed a *Drosophila* RNAi-based screening system.

**Figure 1:**
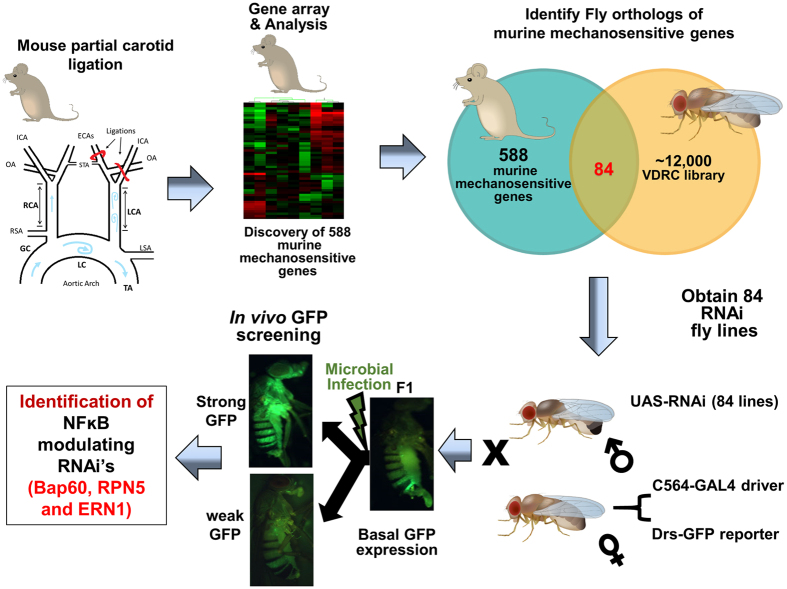
Overall study design and work flow. A previous mRNA microarray study identified 588 mechanosensitive genes from endothelial cells exposed to disturbed blood flow (*d-flow*) using the mouse partial ligation model. Of these 588 mechanosensitive genes, we found 84 corresponding *Drosophila* orthologs. RNAi flies for these genes were obtained and mated with *C564-GAL4* flies containing a *drosomycin-GFP* reporter (*drosomycin-GFP* RNAi flies). *Drosomycin-GFP* expression under both basal and Gram-positive bacterial infection was used to screen the RNAi fly line by stereo fluorescence microscopy. IHJ prepared the illustrations of mouse and *Drosophila* using Adobe Illustrator. SK prepared the illustration for partial carotid ligation model using Microsoft PowerPoint software.

By comparing the *Entrez IDs* of 588 previously identified mechanosensitive murine gene^6^ to fly orthologs (http://flybase.org), we first identified and obtained 84 *Drosophila* RNAi lines carrying *Drosophila* orthologs of murine mechanosensitive genes (Fig. 1; Supplementary Table 1).

### Generation and screening of *Drosophila* RNAi lines expressing *drosomycin-GFP*

We tested the effects of 84 *Drosophila* orthologs of murine mechanosensitive genes on the Toll pathway by using reporter flies carrying *drosomycin-GFP*. To constitutively induce the expression of a specific RNAi, male UAS-RNAi lines were mated with female *C564-GAL4* flies carrying *drosomycin-GFP*^12^,^13^,^15^. The *C564-GAL4* flies that express GAL4 mainly in the fat body (immune tissue in *Drosophila*) were used for tissue-specific knockdown of the target gene^31^. The progeny F1 was used to determine NF-κB/Dif-dependent *drosomycin* expression following Gram-positive bacterial infection condition. We found that expression of six RNAi fly lines in this study showed lethality (Supplementary Table 1), and they were not further studied. Expression of GFP in the remaining 78 RNAi lines was examined by fluorescence stereo microscopy before and after 24 h of Gram-positive bacterial infection as shown by the representative fluorescence images (Supplementary Table 4).

### Identification of *Bap60*, *RPN5* and *ERN1* as regulators of inflammation in *Drosophila*

**Figure 2:**
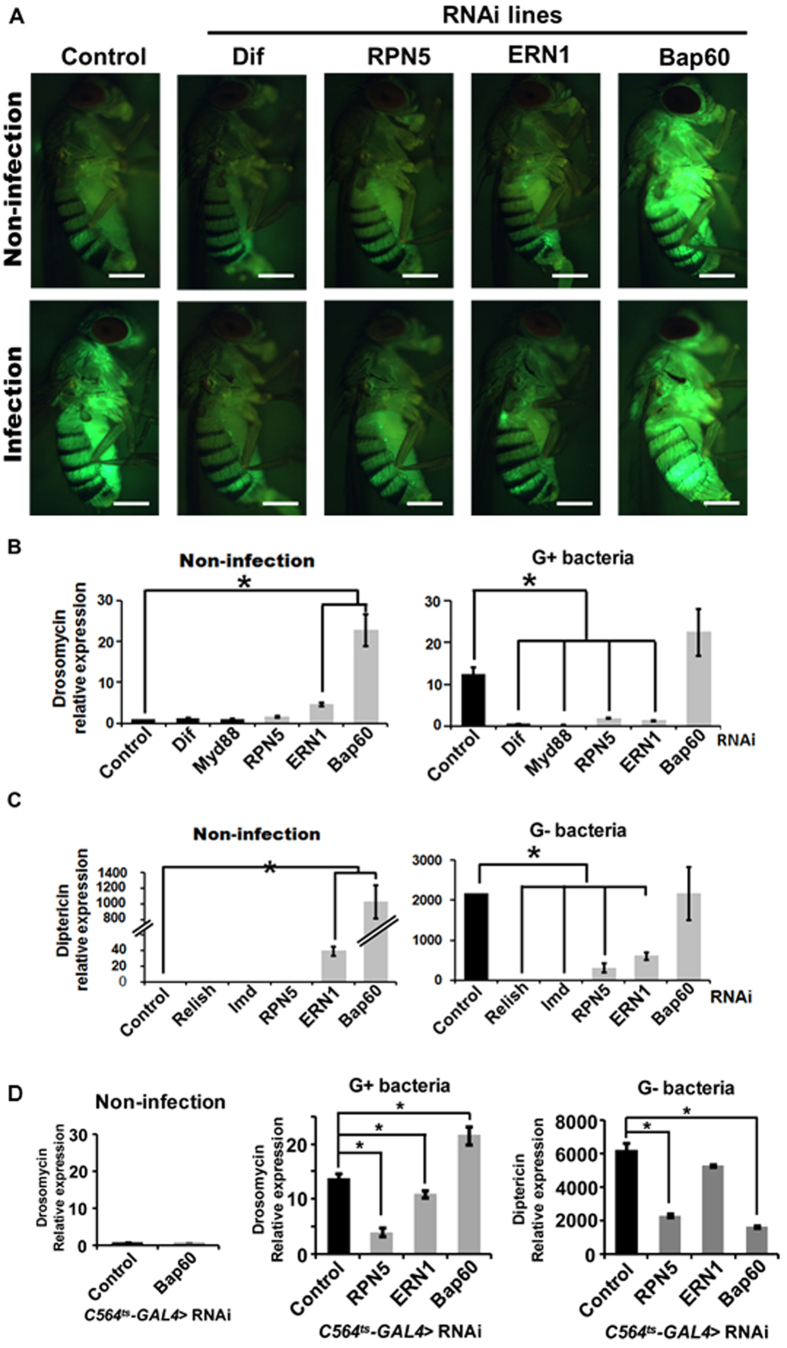
Identification of RNAi lines regulating NF-κB activation in *Drosophila*. **(A)** Stereo fluorescence microscopy was used to monitor the *drosomycin-GFP* expression in each *drosomycin-GFP* RNAi fly line under both basal and Gram-positive bacterial infection. As a control, the *Dif*-RNAi fly was used. GFP expression in three or more independent cohorts comprising ~10 adults each was monitored after 24 h of infection. White Scale Bar = 500μm **(B)** qPCR assay for *drosomycin* mRNA levels in non-infection or in response to Gram positive bacterial infection for 24 h. Dif (a *Drosophila* NF-κB) and Myd88 RNAi flies were used as negative controls. Drosomycin level in the non-infection control was taken arbitrarily as set 1. Values represent the mean ± S.E.M (*P < *0.05) of* at least three independent experiments. **(C)** qPCR assay was carried out for *diptericin* mRNA level, a NF-κB target gene, in uninfected control and in response to Gram negative bacterial infection for 6 h. Relish and IMD RNAi flies were used as negative controls. Diptericin level in non-infection condition was taken arbitrarily as set 1. Values represent the mean ± S.E.M (*P < *0.05) of* at least three independent experiments. **(D)** qPCR assay for *drosomycin* mRNA levels in non-infection condition (left) and Gram-positive bacterial infection for 24 h (middle). Respective RNAi was induced by maintaining the *C564*^*ts*^*-GAL4* flies at 29 °C for 5 days. *Drosomycin* levels in the non-infected control flies was taken arbitrarily as 1. Values represent the means ± S.E.M (*P < *0.05)* of at least three independent experiments. (right panel) qPCR for *diptericin* mRNA expression, an NF-κB target gene, in response to Gram-negative bacterial infection for 6 h was carried out using the *C565*^*ts*^*-GAL4*-driven RNAi flies. *Diptericin* level in non-infection condition of control flies was taken arbitrarily as 1. Values represent the means ± S.E.M (*P < *0.05)* of at least three independent experiments.

From the above initial *in vivo* GFP screening using the constitutive RNAi expression system *(C564-GAL flies)*, we identified three genes, *RPN5*, *ERN1*, and *Bap60*, that substantially modified NF-κB activation (Fig. 2). *Dif* (a *Drosophila* NF-κB pathway gene indispensable for immunity to Gram-positive bacteria) RNAi fly, which was used as a negative control, did not show activation of NF-κB in response to the bacterial infection, as expected (Fig. 2A). *RPN5* RNAi and *ERN1* RNAi blocked NF-ΚB activation in response to bacterial infection. Interestingly, *Bap60* RNAi fly line showed a significantly higher NF-κB activation in basal condition which did not further increase in response to the bacterial infection (Fig. 2A). These results suggestes that *RPN5* and *ERN1* may act as NF-κB activators, while *Bap60* acts as a NF-κB repressor.

Next, we validated these qualitative screening results by determining the expression of *drosomycin* by qPCR using total RNAs extracted from these flies. The qPCR results using the constitutive RNAi expression system *(C564-GAL flies)* showed that *drosomycin* expression was increased by >10-fold by Gram-positive bacterial infection in the control RNAi flies, which was significantly reduced in *RPN5* and *ERN1* RNAi lines (Fig. 2B). Furthermore, *drosomycin* expression was increased by >20-fold over the control (Control) in the *Bap60* RNAi fly line (Fig. 2B). Importantly, Gram-positive bacterial infection did not further increase the *drosomycin* expression in *Bap60* RNAi flies (Fig. 2B). RNAi flies for Dif and Myd88, two well-known signaling molecules mediating Toll-induced NF-κB activation, were used as controls to test the specificity of *drosomycin* expression in this screening system (Fig. 2B). Both *Dif* RNAi and Myd88 RNAi lines showed no significant expression of *drosomycin* in non-infected and as well as Gram-positive bacterial infection conditions. These results further substantiate the GFP fluorescence imaging results above (Fig. 2A), suggesting the potential role of *RPN5* and *ERN1* as NF-κB activators and *Bap60* as NF-κB repressor.

It is well known that the NF-κB pathway in *Drosophila* can be activated by Toll-mediated (by Gram-positive bacteria) and IMD-mediated (by Gram-negative bacteria) pathways^32^. Therefore, we tested whether *RPN5, ERN1* and *Bap60* can also regulate IMD-mediated NF-κB activation as measured by *diptericin* expression in response to Gram-negative bacterial infection using the constitutive RNAi expression system. Upon Gram-negative bacterial infection, *diptericin* expression was increased by >2,000-fold over the non-infected control, which was significantly reduced in *RPN5* and *ERN1* RNAi lines (Fig. 2C). Interestingly, *Bap60* RNAi fly line showed a >1,000-fold increase in the *diptericin* expression in the non-infected condition which was further increased to >2,000-fold 6 hours after Gram-negative bacterial infection (Fig. 2C). In addition, *ERN1* RNAi fly also showed a substantial increase in the *diptericin* expression in the non-infected condition which also increased by Gram-negative bacterial infection.

Since Relish and IMD are two well-known upstream regulators of *diptericin* in the IMD-mediated NF-κB pathway in response to Gram-negative bacterial infection, we examined *diptericin* expression in Relish RNAi and IMD RNAi flies. As expected, both Relish RNAi and IMD RNAi flies showed no significant expression of *diptericin* in non-infected and Gram-negative bacterial infection conditions (Fig. 2C). These results indicated that knockdown of *Bap60* induces NF-κB activation in both the Toll-mediated and IMD-mediated pathways.

Previous studies^33^,^34^ indicate that Bap60 mutants may develop melanotic masses which could result in aberrant activation of NF-κB as we observed in our current study. To address this, we tested the role of Bap60 in an inducible RNAi expression system using the temperature-inducible *C564-GAL4-Tub-GAL80*^*ts*^ *(C564*^*ts*^*-GAL4)* flies. Similar to the constitutive RNAi expression, the inducible knockdown of Bap60 in adult flies resulted in an increased *drosomycin* expression in response to Gram-positive bacterial infection (Fig. 2D). In contrast, unlike the constitutive RNAi flies, temperature-induced knockdown of Bap60 *C564*^*ts*^*-GAL4* flies did not change the basal *drosomycin* expression (Fig. 2D). These results suggest that the increased basal *drosomycin* expression in the constitutive *C564-GAL4*-Bap60-RNAi flies might be related to developmental changes resulting in Toll-mediated NF-κB pathway^35^. However, we failed to observe any obvious signs of melanotic masses in either of the Bap60 RNAi flies in our hands.

Next, we tested whether inducible knockdown of *RPN5*, *ERN1*, and *Bap60* can also regulate Toll- and IMD-mediated NF-κB activation by measuring *drosomycin* and *diptericin* expression, respectively, in Gram-positive and Gram-negative bacterial infection conditions in the C654^*ts*^-Gal4 RNAi flies. First, we found that inducible knockdown of Bap60 did not induce *drosomycin* expression in the basal condition (Fig. 2D), unlike in the constitutive Bap60 knockdown flies (Fig. 2B). However, inducible Bap60 knockdown increased *drosomycin* expression while reducing *diptericin* expression compared to the respective infection controls, suggesting that Bap60 plays a differential role in Toll- and IMD-pathways. In contrast, unlike the constitutive *ERN1* knockdown, the inducible knockdown of *ERN1* showed relatively minor reduction of *drosomycin* expression and no significant effect on *diptericin* expression, in response to respective bacterial infection conditions (Fig. 2D). However, inducible knockdown of *RPN5* showed a significant inhibitory effect on the Toll- and IMD-mediated NF-κB activation (Fig. 2D) as observed in the constitutive *RPN5* RNAi flies (Fig. 2B).

Taken together, by comparing the constitutive and inducible RNAi systems, we have found that *RPN5* (activator) and Bap60 (differential regulator) may be mechanosensitive NF-κB regulators while *ERN1* is a weak NF-κB regulator.

### Smarcd3, a mammalian ortholog of *Bap60*, is a mechanosensitive protein expressed in mouse aortic endothelium

**Figure 3:**
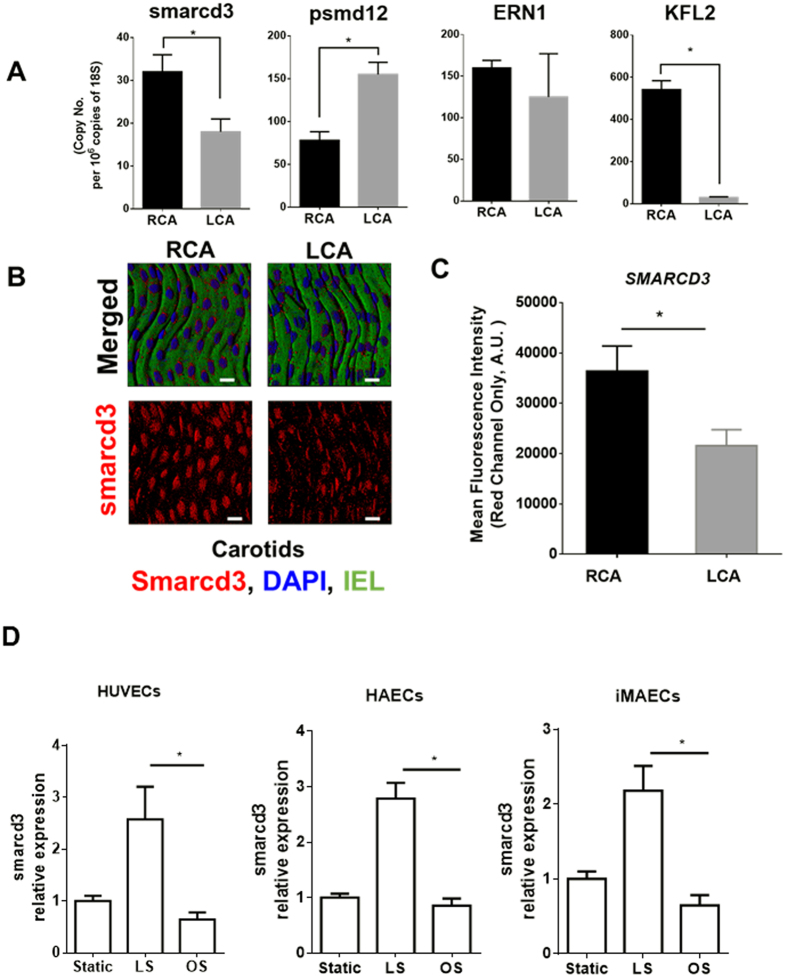
Validation of mechanosensitivity of *smarcd3 (Bap60* ortholog), *psmd12 (RPN5* ortholog) and *ERN1* in mouse arterial endothelium. C57Bl6 mice underwent partial ligation of the LCA and were sacrificed 48 h later. (**A**) Endothelial-enriched RNAs were isolated from RCA and LCA, and the expression of *smarcd3, psmd12* and *ERN1* was determined by qPCR (n = 3, *p < 0.05). *KLF2* was used as a control. (**B**) LCAs and RCAs were dissected out 48 h post ligation and were mounted *en face.* Immunofluorescence confocal microscopy was performed to detect the expression of smarcd3 (red) and DAPI for nuclei (blue) (n = 3). Auto-fluorescence (green) shows internal elastic lamina (IEL). White Scale Bar = 50 μm. (**C**) Image J was used to perform the semi-quantitative estimation of smarcd3 expression in the RCA vs. LCA by calculating the mean fluorescence intensities from the Red Channel. Data shown as means ± S.E.M from 5 different animals; *p < 0.05. Mean fluorescence intensities from the negative control images (Secondary Antibody only) were used for background subtraction. (**D**) Human umbilical vein endothelial cells (HUVECs), human arterial endothelial cells (HAECs), and immortalized mouse aortic endothelial cells (iMAECs) were subjected to either laminar shear (LS) or oscillatory shear (OS) for 24 h. RNA was extracted and expression of *smarcd3* was determined by qPCR (n = 5, p < 0.05). Static cells were used as controls.

We next examined the role of *RPN5, ERN1* and *Bap60* in mouse arterial endothelium. For this, we first examined the mRNA expression of *smarcd3*, *psmd12* (a mammalian ortholog of *RPN5*) and *ERN1*, in order to validate the mechanosensitive regulation of these genes in the mouse arterial endothelium. Using our mouse partial carotid ligation model, we induced *d-flow* in the LCA, while the contralateral RCA continued to experience *s-flow*^16^,^17^. Endothelial-enriched mRNAs obtained from both carotids 48-hour post-ligation showed that expression of *smarcd3* in the LCA endothelium was decreased by approximately 50%, while *psmd12* was increased by 2.2-fold as compared to the RCAs (Fig. 3A). However, the expression of *ERN1* was not statistically different. As a control experiment, we used *KLF2* as a well-known flow-sensitive gene^6^ (Fig. 3A). These findings indicate that smarcd3 and *psmd12* are flow-sensitive genes in endothelial cells, whereas the mechanosensitivity of *ERN1* could not be confirmed.

Next, we validated the mechanosensitivity of smarcd3 by a specific antibody, but not psmd12, as the antibody was unavailable. Consistent with our qPCR analyses, we found that Smarcd3 protein expression in the LCA endothelium (where flow is disturbed and endothelium is inflamed) was significantly reduced as compared to the RCA (where flow is stable and endothelium is not inflamed)^6^,^8^,^17^ (Fig. 3B,C). These results demonstrate that smarcd3 expression is significantly reduced in the mouse arterial endothelium exposed to *d-flow* compared to *s-flow*. Additional *in vitro* experiments using the cone-and-plate shear system also confirms that exposure to the pro-inflammatory oscillatory shear stress (OS) for 24 h decreased the expression of *smarcd3* in HUVECs, HAECs, and iMAECs compared to the anti-inflammatory laminar shear stress (LS) (Fig. 3D). Taken together, these results demonstrate that smarcd3 expression is reduced by the pro-inflammatory *d-flow* condition both *in vitro* and *in vivo*.

### Knockdown of smarcd3 induces endothelial inflammation

**Figure 4:**
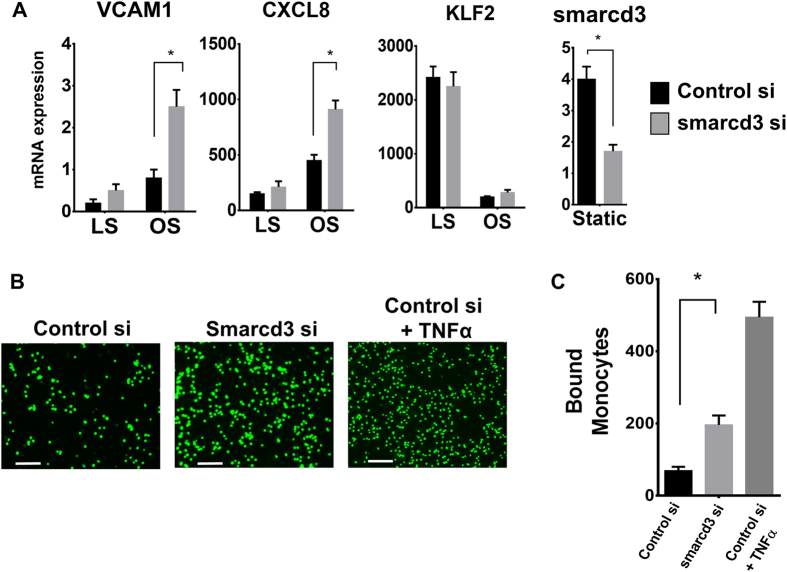
Knockdown of smarcd3 enhances endothelial activation. (**A)** HUVECs were transfected with non-target or *smarcd3-siRNA* (150 nM) for 2 days and were subjected to either LS or OS for 24 h. RNA was prepared and expression of NF-κB target genes (*VCAM1* and *CXCL8*) was determined by qPCR (mean ± S.E.M, n = 4, ^*^p < 0.05). *KLF2* was used as a control. **(B)** HUVECs were transfected with either non-target or *smarcd3*-*siRNA* (150 nM) for 2 days. The number of adhering BCECF-labelled THP1 monocytes (5 × 10^5^) was determined by fluorescence microscopy. As a positive control, some cells were treated with TNFα (3 ng/mL) for 3 hours. White Scale Bar = 200 μm. **(C)** Graph shows quantification of bound monocytes (mean ± S.E.M, n = 3, ^*^p < 0.05).

To determine whether smarcd3 is involved in endothelial inflammation, we treated HUVECs with *smarcd3-siRNA* and subjected them to oscillatory shear conditions. As expected and shown previously^36^,^37^, oscillatory shear induced *VCAM1* and *CXCL8* expression while decreasing the *KLF2* expression compared to laminar shear (Fig. 4A). Knockdown of *smarcd3* further enhanced this oscillatory shear-induced expression of *VCAM1* and *CXCL8* by 3-fold and 1.9-fold, respectively (Fig. 4A). However, knockdown of smarcd3 did not have any effect on the expression of *KLF2* (Fig. 4A). In addition, treatment of *smarcd3-siRNA* in HUVECs significantly increased monocyte adhesion to the endothelium as compared to the control-siRNA (Fig. 4B,C), confirming the role of smarcd3 in endothelial inflammation.

**Figure 5:**
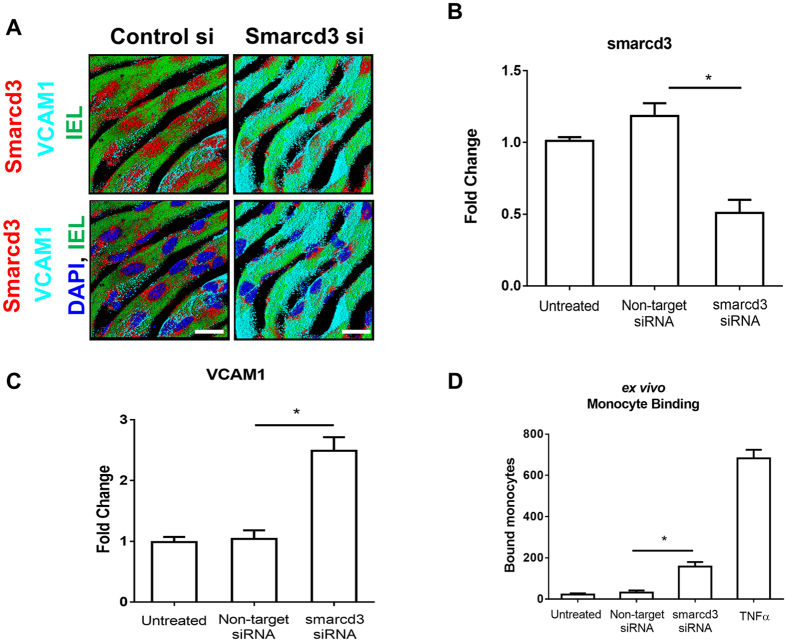
Knockdown of *smarcd3* induces VCAM1 expression in mouse aortic endothelial cells *ex vivo*. **(A)** Mouse aorta explants were cultured *en-face* in a 48-well plate. Aortic explants were transfected with siRNAs (100 nM) for *smarcd3* (si-*Smarcd3*) or control for 48 h and expression of smarcd3 and VCAM1 in the endothelium was determined by immunofluorescence staining using antibodies for smarcd3 (red) and VCAM1 (Cyan). Auto-fluorescence (green) shows internal elastic lamina (IEL). White Scale Bar = 50 μm **(B,C)** Graph shows quantitative estimation of signal intensities for expression of smarcd3 and VCAM1, respectively (mean ± S.E.M, n = 6, ^*^p < 0.05). **(D)** Aortic explants treated as above were mounted *en-face*, and the number of BCECF-labeled J774A.1 monocytic cells to the endothelial surface was determined by fluorescence microscopy. Values represent mean ± S.E.M (*P < 0.05; n = 6).

We next validated the role of smarcd3 in endothelial inflammation by treating mouse aorta explants *ex vivo* with *smarcd3-siRNA*. Treatment of mouse aortic explants with *smarcd3-siRNA* for 48 hours significantly reduced the expression of smarcd3 in the endothelial layer as compared to the non-targeting control [shown by *en-face* immunostaining (Fig. 5A,B)], confirming the efficacy of *smarcd3-siRNA* in our *ex vivo* system. In this condition, we found that knockdown of smarcd3 resulted in increased VCAM1 expression (Fig. 5A,C) and significantly increased monocyte adhesion (Fig. 5D). These results demonstrate that knockdown of smarcd3 induces endothelial inflammation both *in vitro* and *ex vivo*.

## Discussion

Recent technological advances such as microarray and RNA sequencing analyses have enhanced our understanding on a global gene expression pattern during endothelial inflammation and pathogenesis of atherosclerosis. However, it is not easy to individually validate the roles of each gene in the disease progression. Previously, by using a partial carotid ligation model of atherosclerosis, we identified more than 580 mechanosensitive genes that changed in response to *d-flow* as compared to *s-flow*^6^. *D-flow* is known to promote atherosclerosis by regulating global gene expression changes in the endothelium^1^. Although one of the key mechanisms by which *d-flow* causes atherosclerosis is endothelial inflammation^4^, the functions of these flow-sensitive genes during inflammation remain to be elucidated. Here, we developed an *in vivo* functional screening system using the constitutive *Drosophila* RNAi (*C564-GAL4* RNAi) flies that express *drosomycin-GFP* as a surrogate marker of Toll-dependent NF-κB activation. Using this *in vivo* screening strategy, we screened 84 flow-sensitive genes and identified *Bap60*, *RPN5* and *ERN1* as potential regulators of Toll-mediated NF-κB-dependent inflammation pathway. The role of these genes was subsequently validated using the temperature inducible RNAi (*C564*^*ts*^*-GAL4* RNAi) flies to rule out the potential secondary effects of these genes during development. From these fly studies, we identified two mechanosensitive NF-κB regulators: *RPN5* as an activator of both Toll- and IMD-mediated NF-κB pathways, and Bap60 as repressor and activator of Toll-mediated and IMD-mediated NF-κB pathways, respectively. We also identified *ERN1* as a weak activator of the Toll-mediated pathway.

Subsequent studies in mammalian systems validated smarcd3 (mammalian ortholog of *Bap60*) as a novel mechanosensitive repressor of NF-κB-mediated inflammation in mammalian endothelial cells. To our knowledge, this is the first report where a mammalian mechanosensitive transcriptomic dataset has been functionally screened by taking advantage of *Drosophila* UAS-RNAi knockdown library.

Here, Bap60/smarcd3 was identified as a novel mechanosensitive molecule involved in the downregulation of NF-κB-dependent inflammation. Smarcd3 is a SWI/SNF-related matrix-associated actin-dependent regulator of chromatin subfamily D member-3^38^. Smarcd3 is part of the large chromatin remodeling complex that is known to regulate gene transcription by modifying the chromatin structure^38^,^39^,^40^,^41^. As knockdown of *smarcd3* significantly enhanced oscillatory shear-induced pro-inflammatory response as measured by *VCAM1* and *CXCL8* expression (Fig. 4), it is likely that d-flow induces pro-inflammatory gene expression via reducing smarcd3 expression in the endothelial cells.

Recently, Bonnay *et al.* showed that *Drosophila* Bap60 interacts with Akirin upon immune challenge, and Bap60-Akirin complex is required for the induction of a subset of IMD target genes, indicating that Bap60 acts as a positive regulator for IMD-pathway^42^, and is consistent with our result obtained using the inducible Bap60 RNAi flies (Fig. 2D). In contrast, knockdown of Brahma complex component including Bap60 and Brahma is sufficient to enhance the *drosomycin* reporter activity in S2 cells^42^,^43^. Consistent with this, our inducible Bap60 RNAi fly showed increased *drosomycin* expression upon Gram-positive bacterial infection (Fig. 2D). Together, our results and previous studies show that Bap60 could act as a negative and positive regulator for Toll- and IMD-target gene expression, respectively, in adult flies (Fig. 2C,D). Although, it is not known whether shear stress regulated NF-κB is activated via Toll- and/or IMD-mediated pathways, our mammalian studies suggest that smarcd3 acts as a repressor of NF-κB activation in response to shear stress.

Although our *Drosophila* screening system enabled us to screen for candidate mechanosensitive genes involved in NF-κB activation, there are some limitations in our experimental approaches. For example, our primary screening was limited to testing the Toll-mediated NF-κB activation pathway, but not the IMD-mediated NF-κB pathway. Furthermore, our primary screening with constitutive *C564-GAL4* flies seems to have caused false positive results due to potential secondary effects during development. The use of an inducible GAL4 driver such as *C564*^*ts*^*-GAL4* together with a dual reporter system [by the *drosomycin-GFP* reporter with an IMD-pathway reporter such as *diptericin*-mCherry^44^] would have circumvented these technical limitations. Due to lack of proper reagents, the role of *RPN5* could not be studied.

In summary, we developed an *in vivo Drosophila* RNAi screening method to identify flow-sensitive genes that regulate endothelial inflammation. The newly identified mechanosensitive *smarcd3* may play a critical role in endothelial inflammation and atherosclerosis. This *in vivo* functional screening approach may be expanded to identify novel genes that regulate other pathways such as apoptosis and cell proliferation.

## Additional Information

**How to cite this article**: Kumar, S. *et al.* Functional screening of mammalian mechanosensitive genes using *Drosophila* RNAi library– *Smarcd3/Bap60* is a mechanosensitive pro-inflammatory gene. *Sci. Rep.* **6**, 36461; doi: 10.1038/srep36461 (2016).

**Publisher’s note**: Springer Nature remains neutral with regard to jurisdictional claims in published maps and institutional affiliations.

## Supplementary Material

Supplementary Information
