# Supplementary material for: Functional screening of mammalian mechanosensitive genes using Drosophila RNAi library– Smarcd3/Bap60 is a mechanosensitive pro-inflammatory gene
Source: Sci Rep. 2016 Nov 7;6:36461. doi: 10.1038/srep36461 (PMC5098218; doi:10.1038/srep36461)
Supplement: Supplementary Information [file srep36461-s1.pdf]

SUPPLEMENTARY INFORMATION

Functional screening of mammalian mechanosensitive genes using *Drosophila* RNAi library– *Smarcd3/Bap60* is a mechanosensitive pro-inflammatory gene.

Sandeep Kumar[1]*, In-hwan Jang[1]*, Chan Woo Kim[1], Dong-Won Kang[1], Won Jae Lee[2], and Hanjoong Jo[1,3 ¶]

[1]Wallace H. Coulter Department of Biomedical Engineering, Georgia Institute of Technology and Emory University, Atlanta, GA, USA, [2]National Creative Research Initiative Center for Hologenomics, and School of Biological Sciences, Seoul National University, Seoul 151-742, South Korea, and [3]Division of Cardiology, Department of Medicine, Emory University, Atlanta, GA, USA.

Supplementary Table 1

List of RNAi fly lines showing Drosophila transformant ID and CG numbers with corresponding mouse gene IDs

S. No. (In this study)	Transformant ID	CG Number	Mouse Gene	Illumina Gene_ID	lethality (c564> RNAi)
1	100259	CG8933	Pbx1	ILMN_2913089	
2	100269	CG3724	Pgd	ILMN_3052632	
3	100688	CG32190	Nucb1		
4	100728	CG3209	Agpat6	ILMN_2495363	
5	101340	CG1938	Dync1li2	ILMN_3120652	
6	101403	CG1265	Pqlc3	ILMN_1238215	
7	101960	CG7142	klk10-3	ILMN_2507890	
8	102014	CG14808	Sgcd	ILMN_2633062	
9	103038	CG9796	Ifi30	ILMN_2432550	
10	103249	CG15101	Ephx1	ILMN_1219712	
11	103485	CG8946	Sgpl1	ILMN_2756665	
**12**	**103541**	**CG1100**	**Psmd12**	**ILMN_1225988**	
13	103581	CG31992	Tnrc6c	ILMN_2935386	
14	103631	CG9325	Add1	ILMN_2674367	
**15**	**103634**	**CG4303**	**Smarcd3**	**ILMN_2559943**	
16	103911	CG2875	Noc4l	ILMN_2761918	
17	104386	CG11121	Six2	ILMN_3013874	
18	104575	CG1542	Ebna1bp2	ILMN_1258788	lethal
19	105111	CG11059	Clstn1	ILMN_2718801	
20	105149	CG10565	Dnajc2	ILMN_1257077	
21	105161	CG8362	Nme7	ILMN_2685023	
22	105269	CG16996	klk10-2	ILMN_2507890	
23	105361	CG9096	Ccnd2	ILMN_3144575	
24	105383	CG6721	Rasa3	ILMN_1228031	
25	105403	CG9135	Rcc2	ILMN_2513570	
26	105408	CG4003	Ruvbl1	ILMN_3150811	lethal
27	105492	CG15669	Ndrg3	ILMN_2669404	
28	105604	CG3943	Serhl	ILMN_1248397	
29	105663	CG4755	Arhgap17	ILMN_2474515	
30	105853	CG9224	Chrd	ILMN_1248604	
31	106120	CG4233	Got2	ILMN_2599008	
32	106122	CG31999	Fbln2	ILMN_2993109	
33	106129	CG5033	Bop1	ILMN_2614380	

34	106236	CG8542	Hspa9	ILMN_2727687	lethal
35	106470	CG2789	Tspo	ILMN_1254513	
36	106642	CG9375	Kras	ILMN_1217606	
37	106761	CG9195	Scamp1	ILMN_2622354	
38	106774	CG5408	Trib2	ILMN_1215085	
39	106924	CG1837	Txndc5	ILMN_2678355	
40	107131	CG10465	Kctd10	ILMN_1240256	
41	107176	CG5020	Clip1	ILMN_2833781	
42	107181	CG5497	Mrps28	ILMN_3115796	
43	107190	CG8841	C630004H02Rik	ILMN_2767918	
44	107304	CG3780	Sf3b4	ILMN_1243249	lethal
45	107314	CG3924	Ldb2	ILMN_2891646	
46	107327	CG11711	2700078K21Rik	ILMN_2547840	
47	107590	CG5336	Elmo1	ILMN_2624451	
48	107995	CG2219	Arl4a	ILMN_2666487	
49	108069	CG6218	Nagk	ILMN_2920849	
50	108082	CG5319	Btbd3	ILMN_1226606	
51	108442	CG10724	Wdr1	ILMN_2627179	
52	108449	CG14100	Rnmtl1	ILMN_2618408	
53	108542	CG32758	Snx27	ILMN_1217928	
54	108597	CG4849	Eftud2	ILMN_2757019	lethal
55	108658	CG3723	Dnahc17	ILMN_1249888	
56	108723	CG10728	Wdr77	ILMN_1242787	
57	108773	CG16997	Klk10-1	ILMN_2507890	
58	108840	CG14788	Lsg1	ILMN_2633897	
59	108850	CG3730	Prmt5	ILMN_2706853	
60	109421	CG6136	Cutc	ILMN_2664224	
61	109454	CG4637	dhh		
62	109603	CG5355	Prep	ILMN_2759079	
63	109738	CG6891	Cotl1	ILMN_2598103	
64	109762	CG2173	Ddx27	ILMN_3137804	lethal
65	110321	CG10480	Rcc1	ILMN_1217742	
66	110400	CG8314	Zdhhc3	ILMN_2607880	
67	110454	CG1333	Ero1lb	ILMN_2715558	
68	110682	CG9155	Myo1c	ILMN_2697760	
69	110691	CG4684	Fchsd2	ILMN_1248139	
70	12762	CG6854	Ctps	ILMN_2788593	
71	18873	CG10236	Lama5	ILMN_1221817	
72	22125	CG5433	Klc1	ILMN_2834370	
73	23425	CG5708	Lmo4	ILMN_1224866	
74	25909	CG31917	Znhit1	ILMN_1215879	
75	29096	CG9296	Pde6d	ILMN_2504268	
76	3449	CG31937	Dhrs7	ILMN_1236290	

77	34621	CG3454	Hdc	ILMN_2672190
78	37816	CG10692	Mt2	
79	37823	CG10388	Hoxa7	ILMN_2759563
**80**	**39562**	**CG4583**	**ERN1**	**ILMN_2878071**
81	45300	CG5669	Sp4	ILMN_3089584
82	6446	CG8804	Ppap2a	ILMN_2743013
83	7577	CG11576	2310046K01Rik	ILMN_2669441
84	8209	CG16868	Cachd1	ILMN_2699531

Supplementary Table 2

<u>List and sequences of qPCR primers for mRNA expression (Drosophila)</u>

Primer	Sequence
Drosomycin_Fw	5'-GCAGATCAAGTACTTGTTCGCCC-3'
Drosomycin_Rv	5'-CTTCGCACCAGCACTTCAGACTGG-3'
Diptericin Fw	5'-GGCTTATCCGATGCCCGACG-3'
Diptericin Rv	5'-TCTGTAGGTGTAGGTGCTTCC-3'
Rp49_Fw	5'-AGATCGTGAAGAAGCGCACCAAG-3'
Rp49_Rv	5'-CACCAGGAACTTCTTGAATCCGG-3'

Supplementary Table 3

List and sequences of qPCR primers for mRNA expression (Mouse/Human)

Primer	Sequence
m KLF2-Fw	5’-CCAACTGCGGCAAGACCTAC-3’
m KLF2-Rv	5’-AGTCGACCCAGGCTACATGTG-3’
m smarcd3_Fw	5’-CATGGAATTCGAACCCAAACA-3’
m smarcd3_ Rv	5’-TTTCGGAGCAGGAAAGCCCTGG-3’
m psmd12_Fw	5’- ACATGGTGTCCACGTCCCGCA -3’
m psmd12_ Rv	5’-TCGCAGCTTGACTGGCAGGTCT-3’
m ERN1_Fw	5’-TTGAGGAATTACTGGCTTCTCAT-3’
m ERN1_ Rv	5’-TCCAGCATCTTGGTGGATG-3’
hm 18s_Fw	5’-AGGAATTGACGGAAGGGCACCA-3’
hm 18s_ Rv	5’-GTGCAGCCCCGGACATCTAAG-3’
h KLF2-Fw	5’-AGACCTACACCAAGAGTTCGCATC-3’
h KLF2-Rv	5’-CATGTGCCGTTTCATGTGCAGC-3’
h CXCL8_Fw	5’-CATGACTTCCAAGCTGGCCG-3’
h CXCL8_Rv	5’-TTTATGAATTCTCAGCCCTC-3’
h Smarcd3_Fw	5’-GGAGGAAGATGGCTGACAAA-3’
h Smarcd3_ Rv	5’-GCCAAGAGGTCCATGTAAGC-3’
h VCAM1_Fw	5’-CATGGAATTCGAACCCAAACA-3’
h VCAM1_ Rv	5’-TTTCGGAGCAGGAAAGCCCTGG-3’

Supplementary Table 4

Visual Scoring of GFP fluorescence Intensity on a scale of 0 (least) to 5(most) at basal and post Gram-positive bacterial infection in control flies and RNAi fly lines with corresponding mouse gene IDs.

#	CG Number	***RNAi*** **Fly Line**	**Whether embryonic Lethal?**	**Representative Image of the fly under basal (Non-infection) condition**	**Visual GFP Scoring**<br><br>**Basal (Non-infection) condition**	**Representative Image of the fly (Post Gram-positive infection condition)**	**Visual GFP Scoring**<br><br>**Post Gram-positive infection**
I		C564		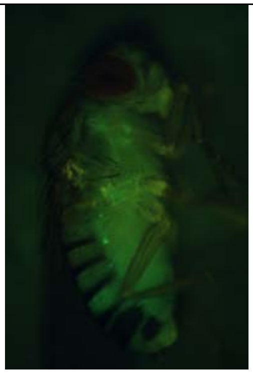	0 ±0	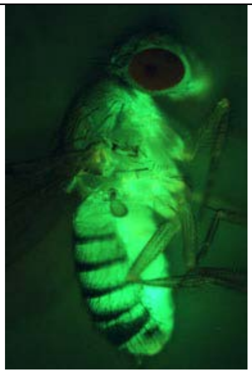	5 ±0
II		Dif		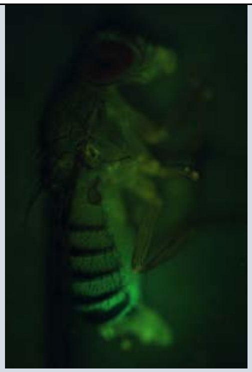	0.3 ±0.3	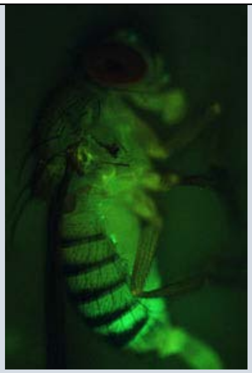	0.4 ±0.24

II		Myd88		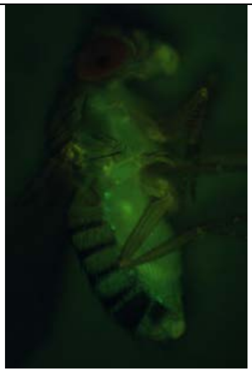	0 ±0	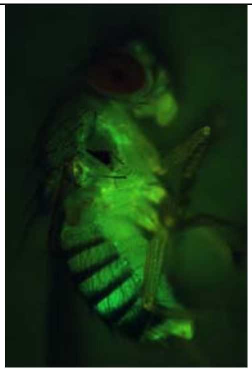	0.4 ±0.24
1	CG8933	Pbx1		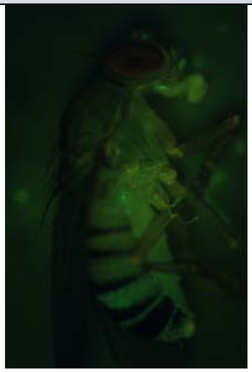	0 ±0	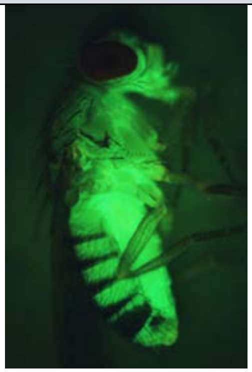	5 ±0
2	CG3724	Pgd		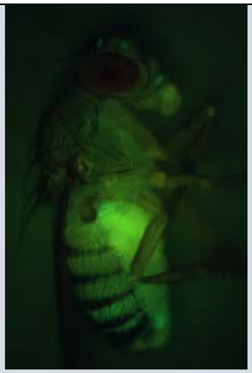	1.2 ±0.5	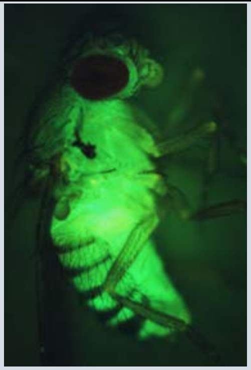	5 ±0

3	CG32190	Nucb1		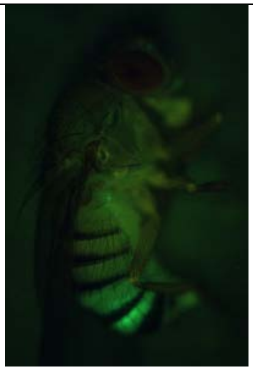	0.3 ±0.1	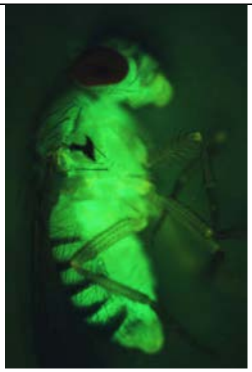	5 ±0
4	CG3209	Agpat6		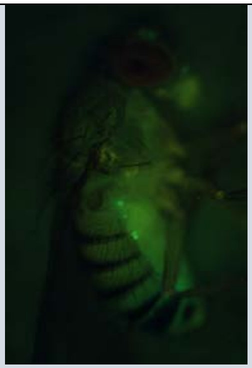	0.3 ±0.3	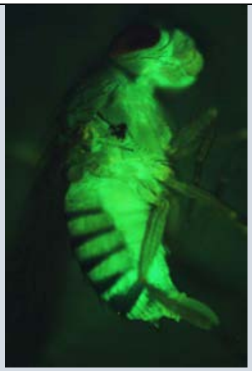	4.9 ±0.13
5	CG1938	Dync1li2		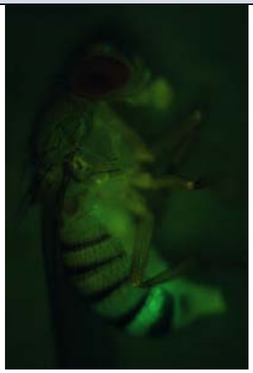	0.3 ±0.3	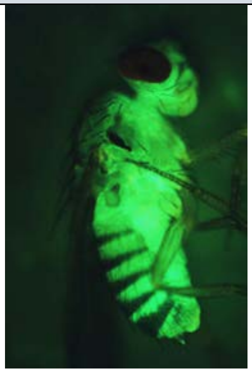	5 ±0

6	CG1265	Pqlc3		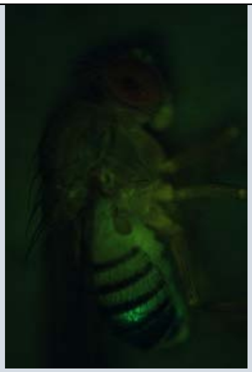	0 ±0	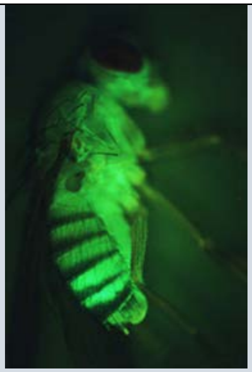	5 ±0
7	CG7142	klk10-3		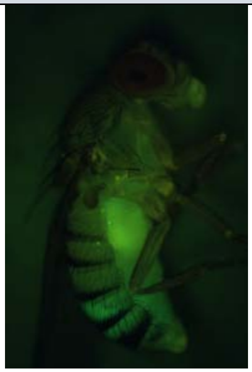	0.8 ±0.4	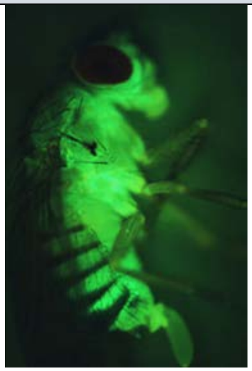	5 ±0
8	CG14808	Sgcd		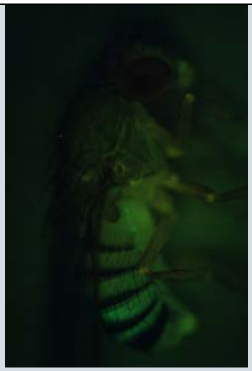	0 ±0	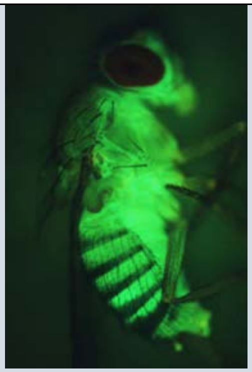	5 ±0

9	CG9796	Ifi30		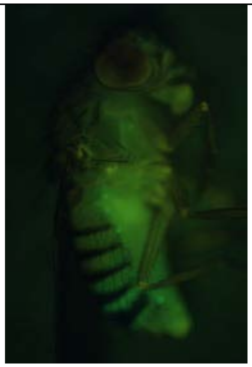	0.4 ±0.4	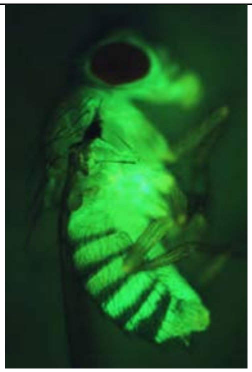	4.9 ±0.13
10	CG15101	Ephx1		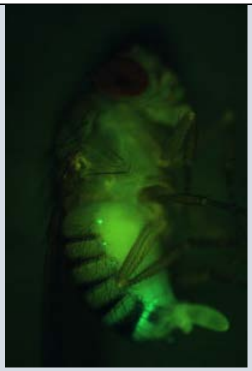	1.4 ±0.9	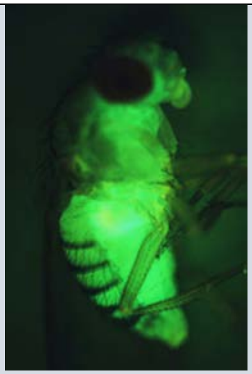	5 ±0
11	CG8946	Sgpl1		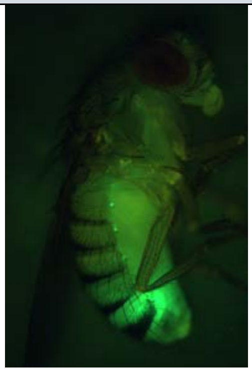	1 ±0.4	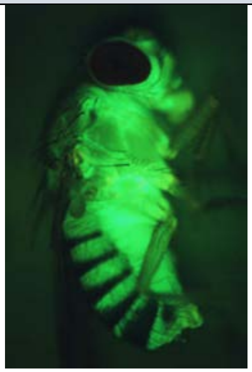	5 ±0

**12**	**CG1100**	**Psmd12**		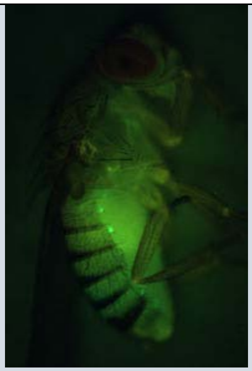	**1.4 ±0.9**	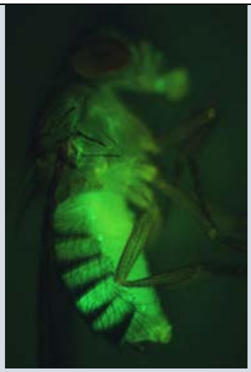	**3.7 ±0.56**
13	CG31992	Tnrc6c		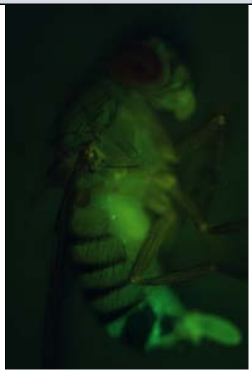	0.3 ±0.3	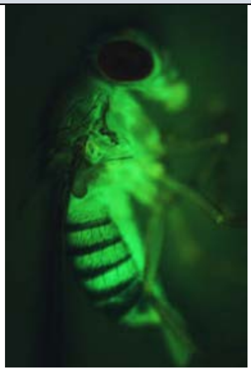	4.5 ±0.21
14	CG9325	Add1		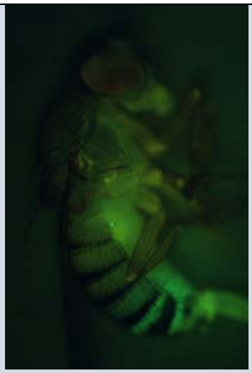	0.3 ±0.3	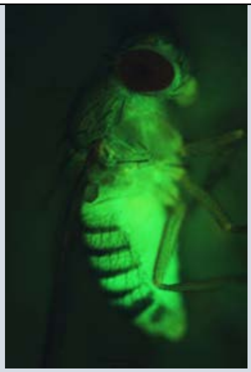	4.8 ±0.25

**15**	**CG4303**	**Smarcd3**		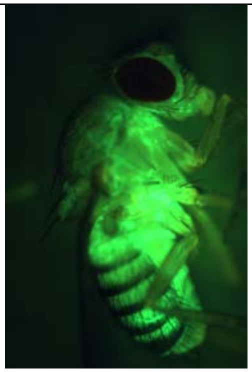	**3.5 ±1.2**	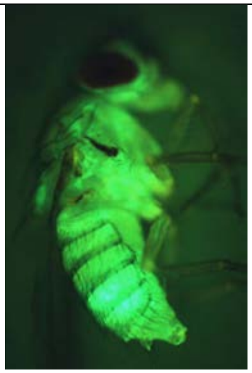	**5 ±0**
16	CG2875	Noc4l		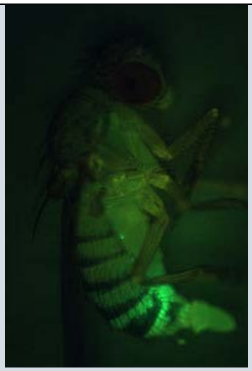	0.2 ±0.1	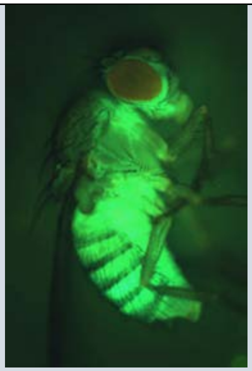	5 ±0
17	CG11121	Six2		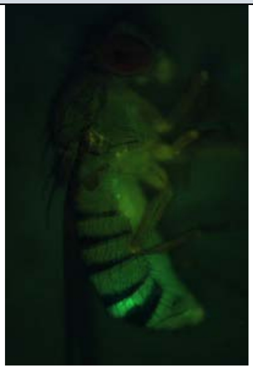	0.2 ±0.1	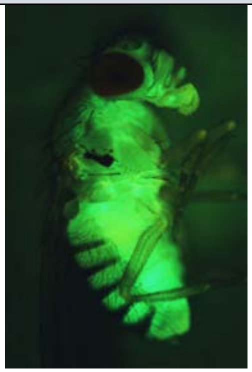	5 ±0
18	CG1542	Ebna1bp2	lethal		NA		NA

19	CG11059	Clstn1		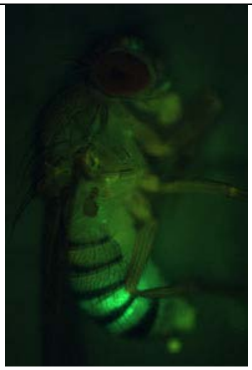	0.5 ±0.2	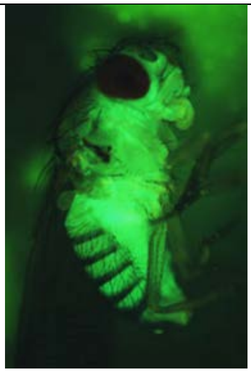	5 ±0
20	CG10565	Dnajc2		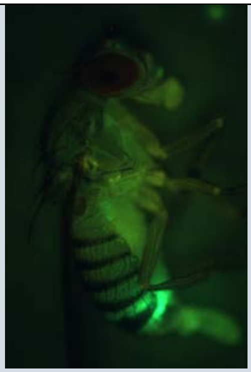	0.8 ±0.6	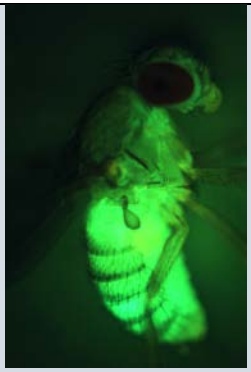	5 ±0
21	CG8362	Nme7		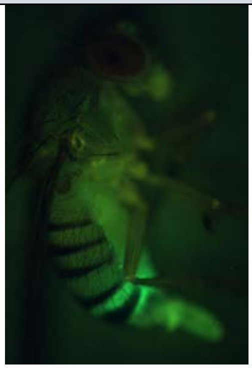	0.8 ±0.6	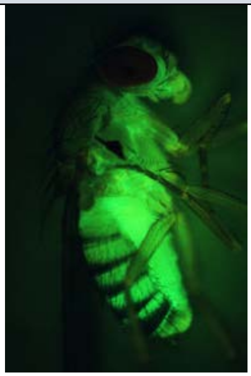	5 ±0

22	CG16996	klk10-2		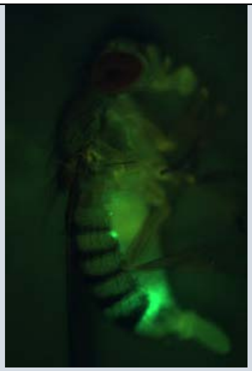	1.2 ±0.6	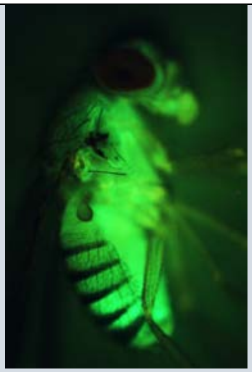	5 ±0
23	CG9096	Ccnd2		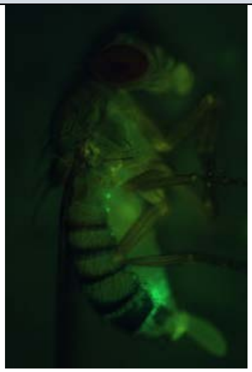	0.7 ±0.6	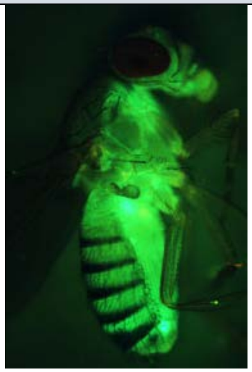	5 ±0
24	CG6721	Rasa3		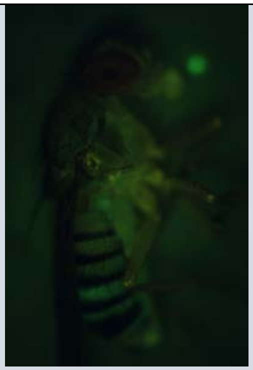	0 ±0	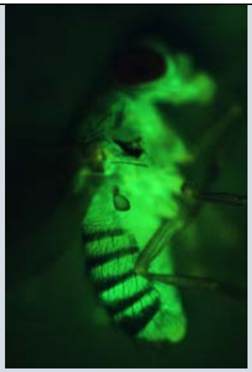	3.9 ±0.43

25	CG9135	Rcc2		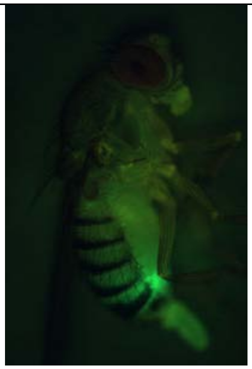	0.4 ±0.4	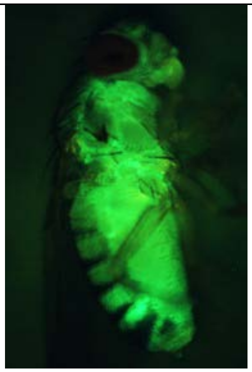	4.5 ±0.36
26	CG4003	Ruvbl1	lethal		NA		NA
27	CG15669	Ndrg3		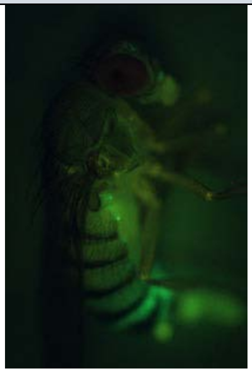	0.3 ±0.3	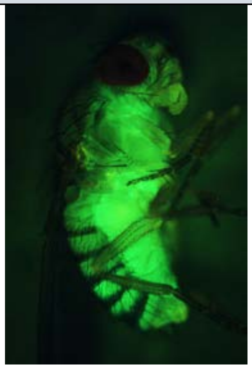	5 ±0
28	CG3943	Serhl		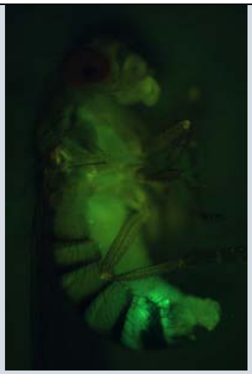	0.4 ±0.4	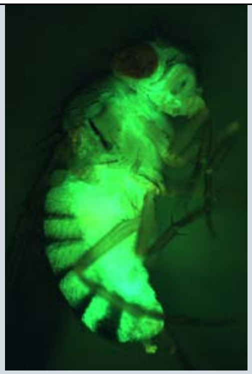	5 ±0

29	CG4755	Arhgap17		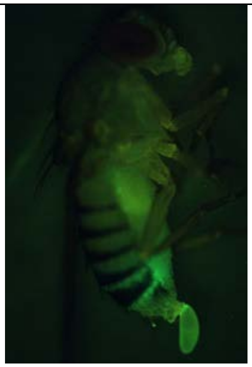	0.3 ±0.3	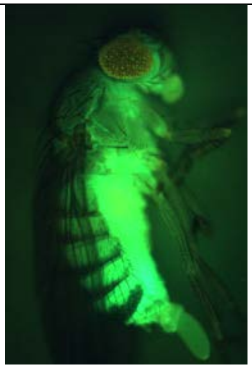	5 ±0
30	CG9224	Chrd		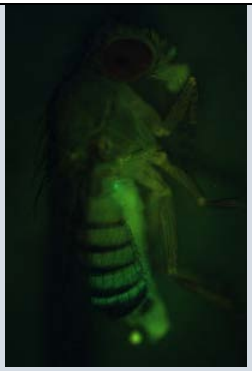	0.3 ±0.3	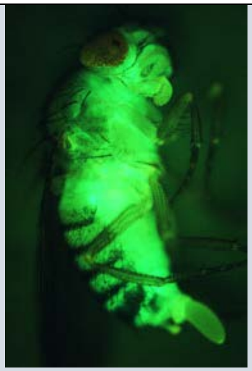	4.4 ±0.24
31	CG4233	Got2		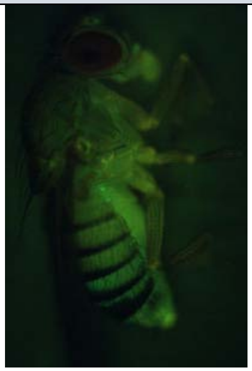	0.4 ±0.4	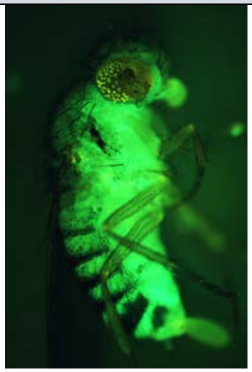	5 ±0

32	CG31999	Fbln2		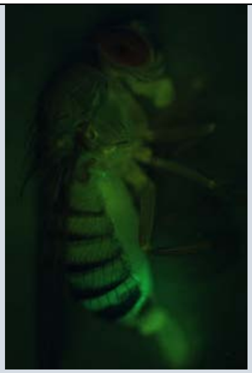	0.2 ±0.1	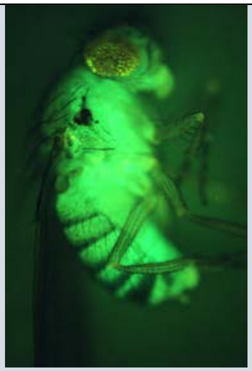	4.8 ±0.25
33	CG5033	Bop1		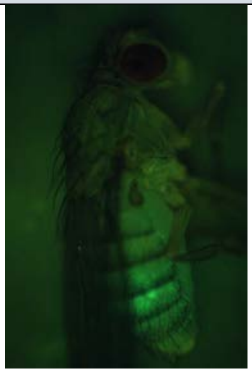	0.2 ±0.1	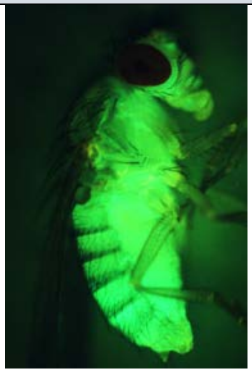	5 ±0
34	CG8542	Hspa9	lethal		NA		NA
35	CG2789	Tspo		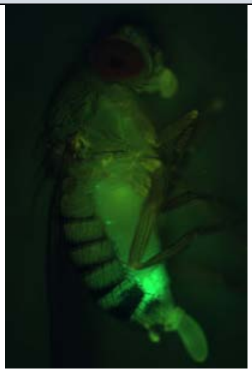	1 ±0.4	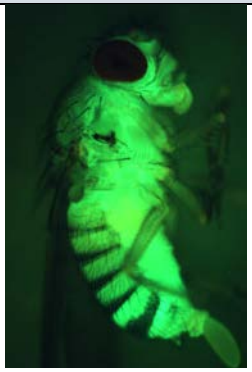	5 ±0

36	CG9375	Kras		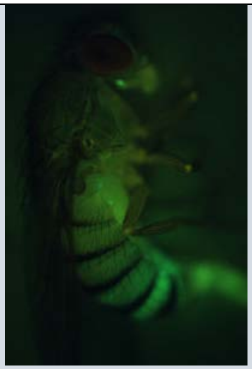	0.4 ±0.4	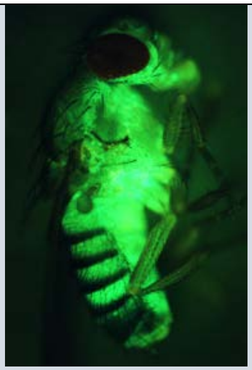	5 ±0
37	CG9195	Scamp1		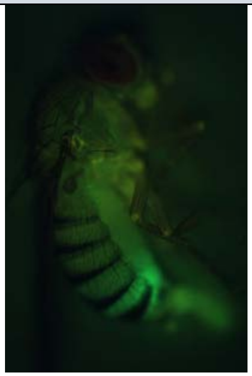	0.3 ±0.3	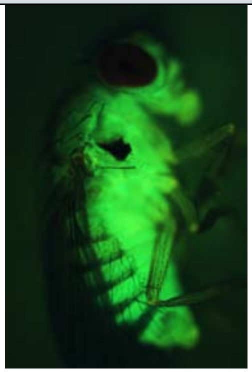	5 ±0
38	CG5408	Trib2		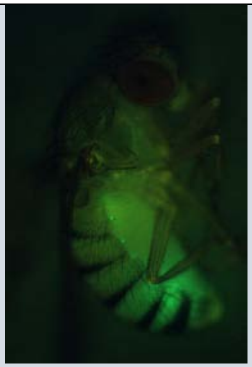	0.8 ±0.6	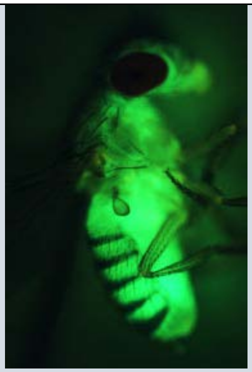	4.9 ±0.13

39	CG1837	Txndc5		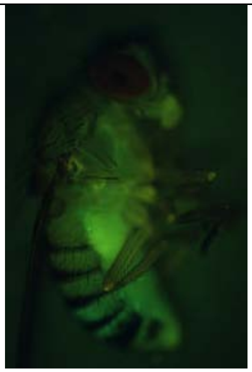	0.5 ±0.5	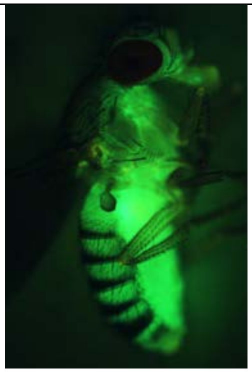	5 ±0
40	CG10465	Kctd10		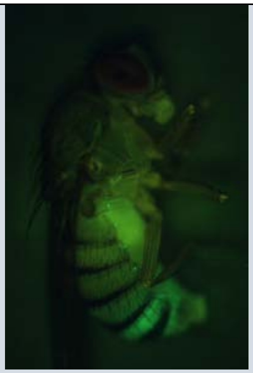	0.7 ±0.5	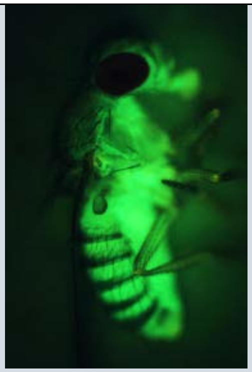	4.8 ±0.25
41	CG5020	Clip1		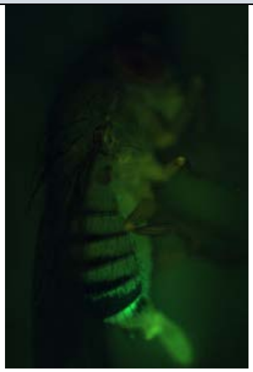	0.2 ±0.1	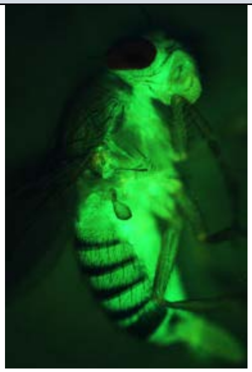	4.4 ±0.24

42	CG5497	Mrps28		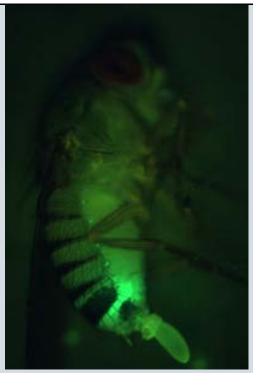	0.8 ±0.3	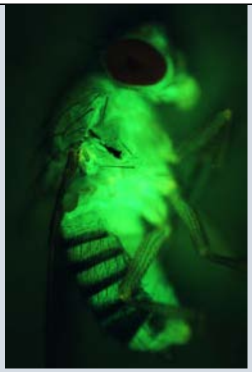	4.5 ±0.29
43	CG8841	C630004H02Rik		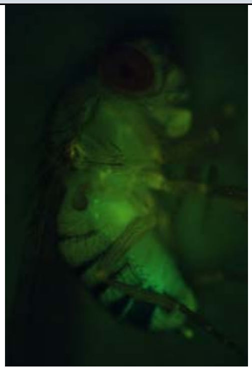	0.5 ±0.3	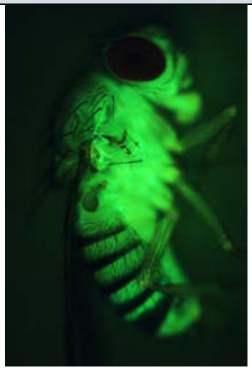	4.8 ±0.25
44	CG3780	Sf3b4	lethal		NA		NA
45	CG3924	Ldb2		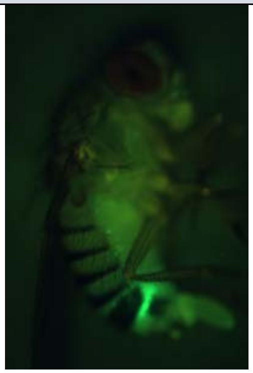	0.4 ±0.4	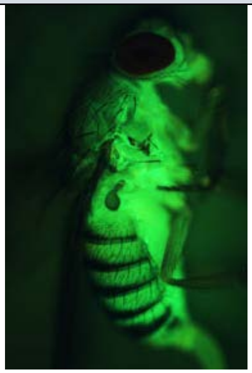	4.9 ±0.13

46	CG11711	2700078K21Rik		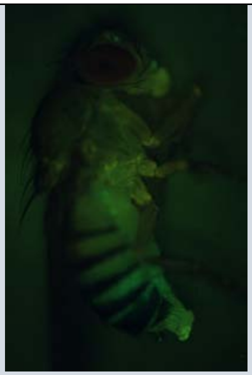	0 ±0	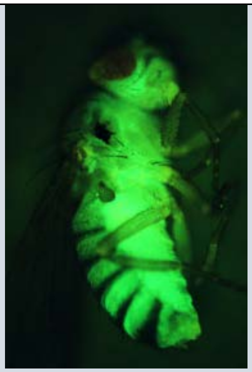	4.8 ±0.25
47	CG5336	Elmo1		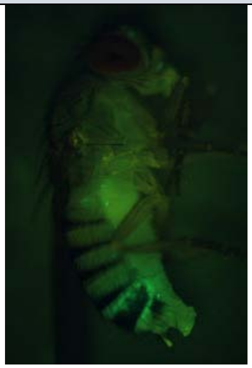	0.2 ±0.1	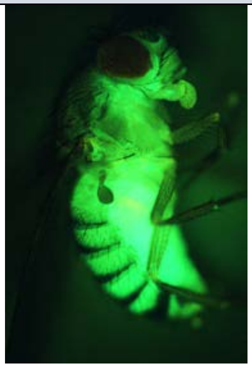	4.2 ±0.52
48	CG2219	Arl4a		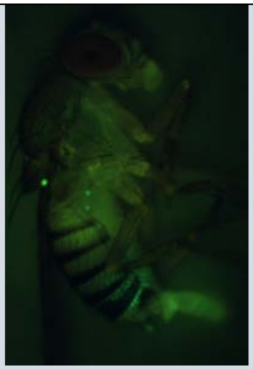	0.4 ±0.4	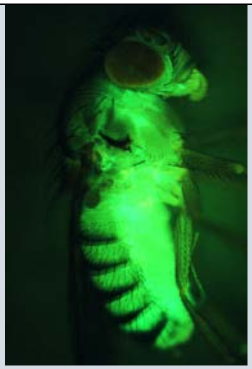	4.8 ±0.25

49	CG6218	Nagk		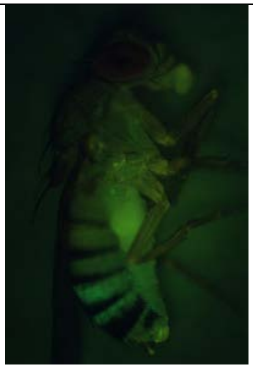	0.2 ±0.1	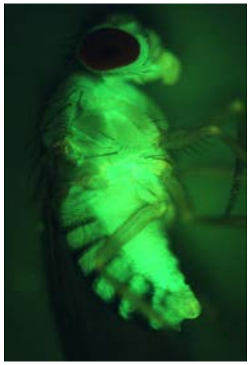	4.8 ±0.25
50	CG5319	Btbd3		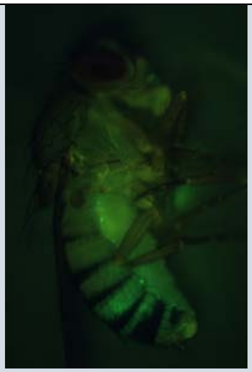	0 ±0	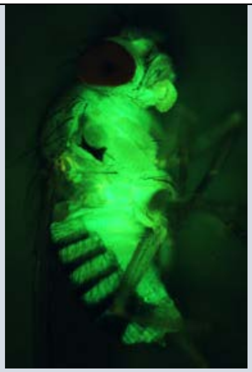	4.8 ±0.25
51	CG10724	Wdr1		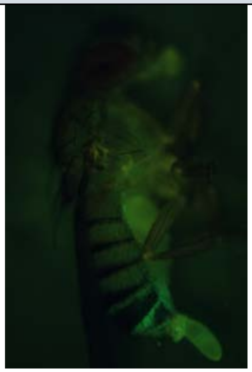	0.4 ±0.4	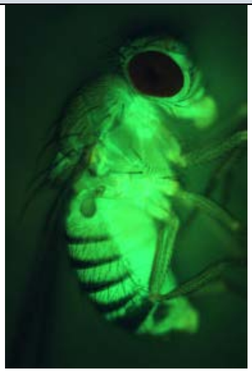	4.2 ±0.43

52	CG14100	Rnmtl1		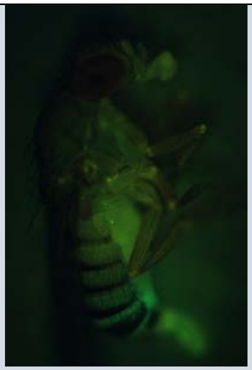	0.2 ±0.1	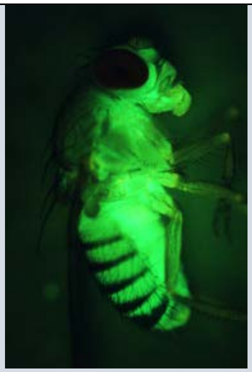	4.8 ±0.25
53	CG32758	Snx27		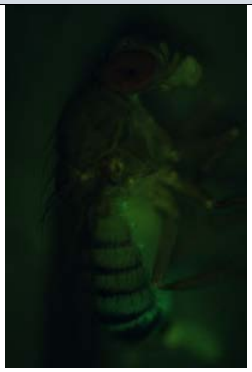	0 ±0	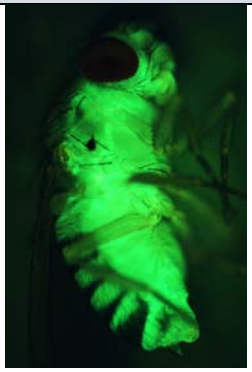	4.3 ±0.48
54	CG4849	Eftud2	lethal		NA		NA
55	CG3723	Dnahc17		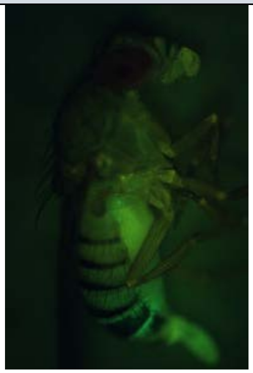	0.4 ±0.2	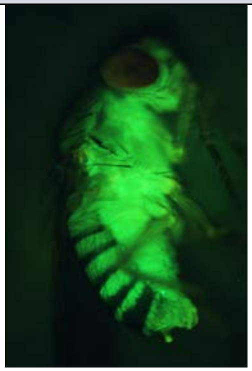	4.8 ±0.25

56	CG10728	Wdr77		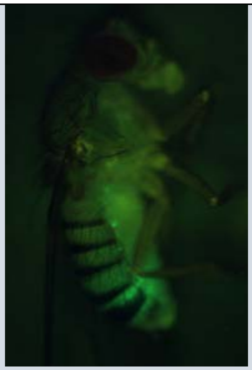	0.3 ±0.1	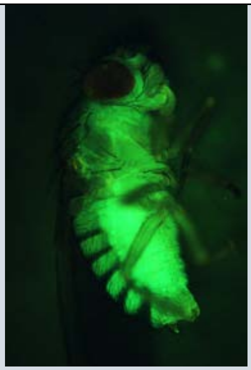	4.9 ±0.13
57	CG16997	Klk10-1		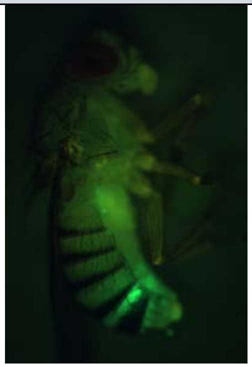	0.4 ±0.2	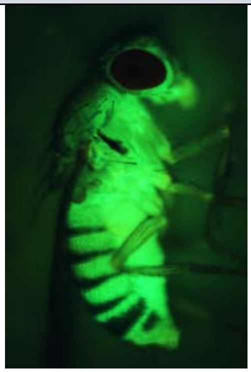	4.8 ±0.25
58	CG14788	Lsg1		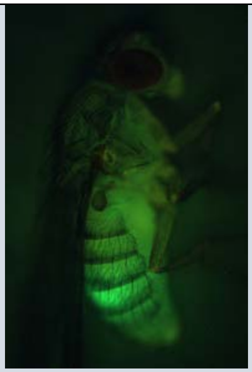	1.2 ±0.5	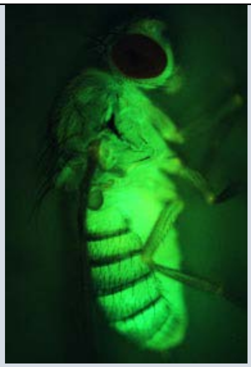	4.8 ±0.25

59	CG3730	Prmt5		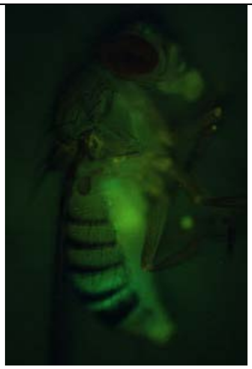	0.7 ±0.5	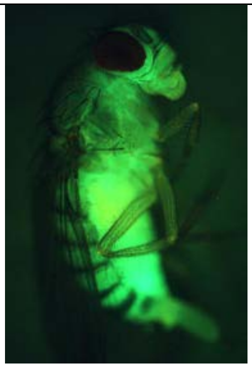	4.8 ±0.25
60	CG6136	Cutc		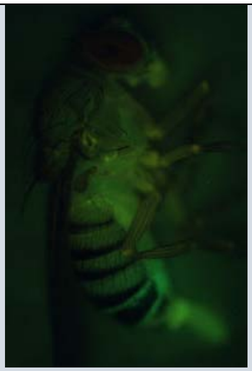	0.2 ±0.1	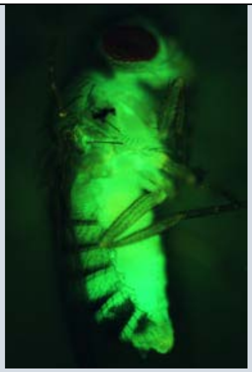	4.7 ±0.24
61	CG4637	dhh		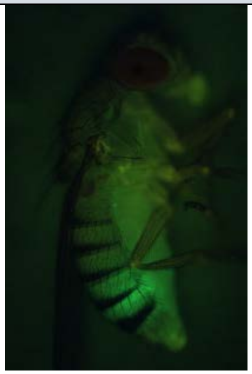	0.7 ±0.4	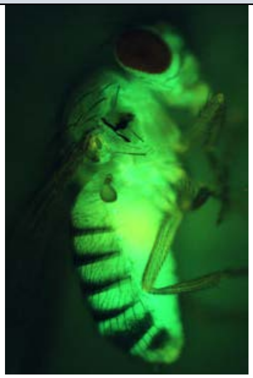	5 ±0

62	CG5355	Prep		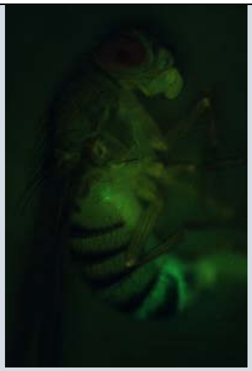	0.2 ±0.1	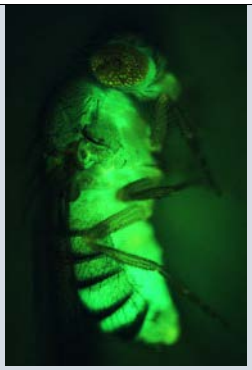	5 ±0
63	CG6891	Cotl1		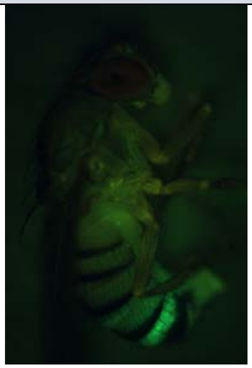	0 ±0	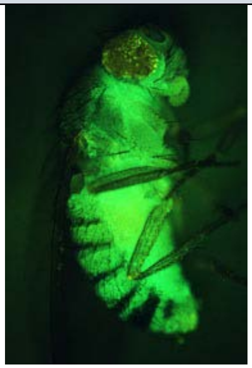	5 ±0
64	CG2173	Ddx27	lethal		NA		NA
65	CG10480	Rcc1		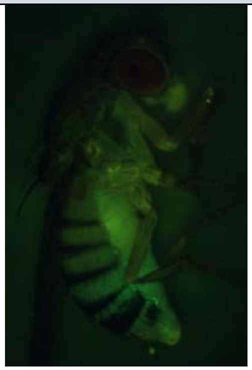	0.2 ±0.1	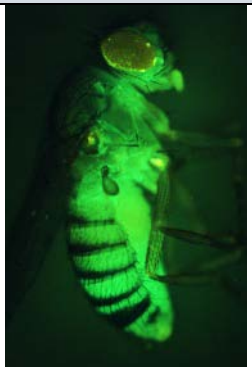	4.5 ±0.29

66	CG8314	Zdhhc3		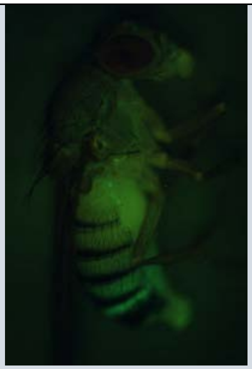	0.2 ±0.1	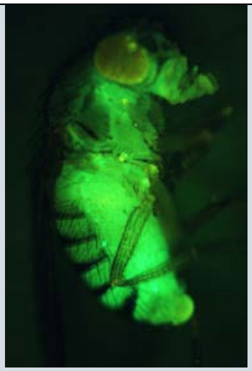	4.9 ±0.13
67	CG1333	Ero1lb		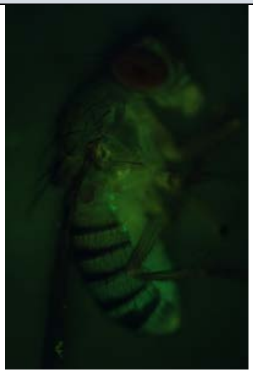	0 ±0	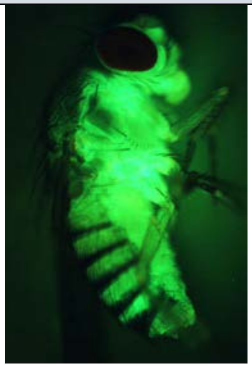	5 ±0
68	CG9155	Myo1c		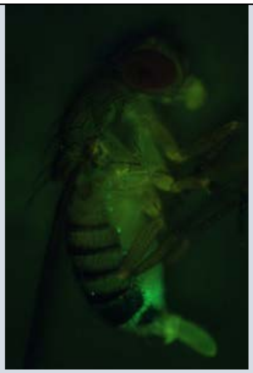	0.5 ±0.3	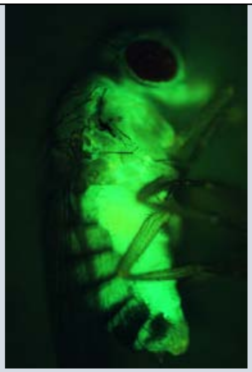	5 ±0

69	CG4684	Fchsd2		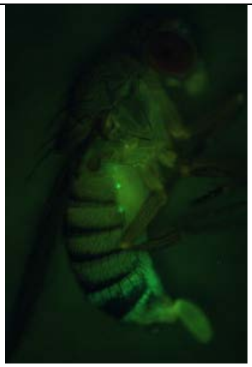	0.3 ±0.3	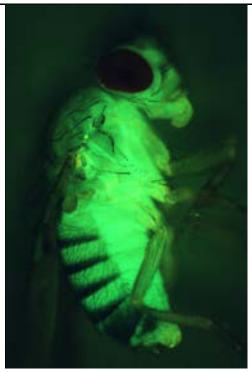	5 ±0
70	CG6854	Ctps		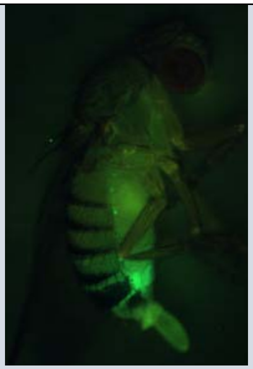	0.7 ±0.4	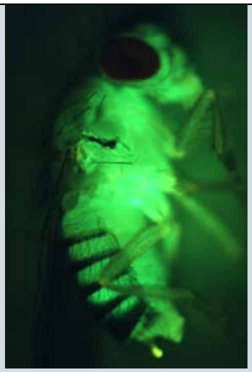	5 ±0
71	CG10236	Lama5		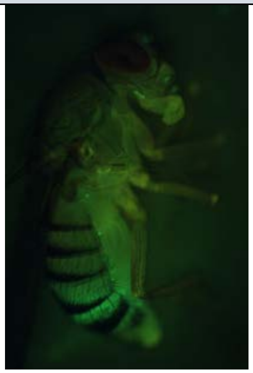	0.4 ±0.4	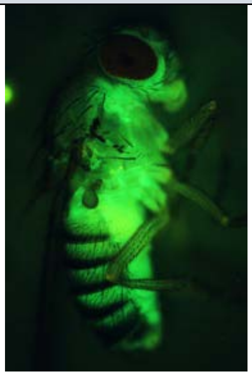	4.5 ±0.21

72	CG5433	Klc1		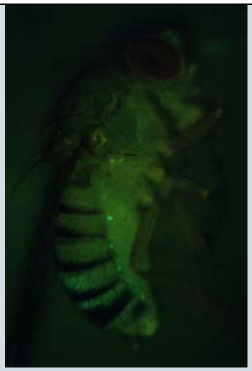	0.2 ±0.1	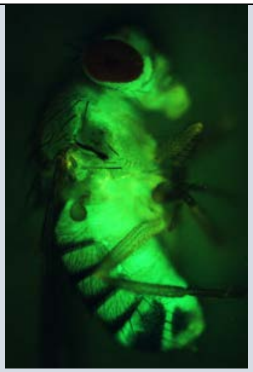	4.8 ±0.15
73	CG5708	Lmo4		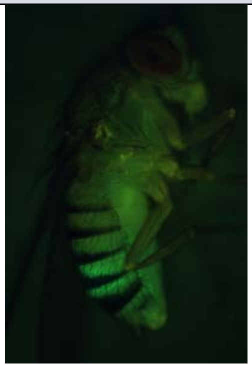	0.4 ±0.4	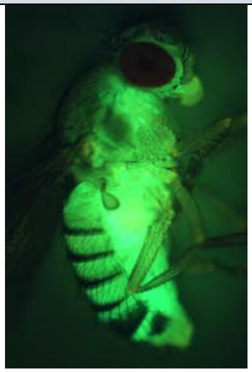	4.9 ±0.13
74	CG31917	Znhit1		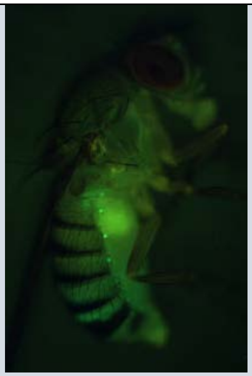	1.5 ±0.5	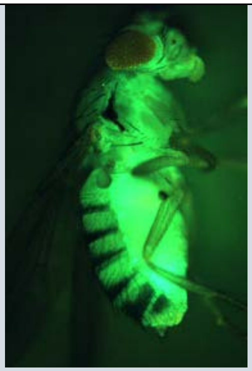	4.8 ±0.25

75	CG9296	Pde6d		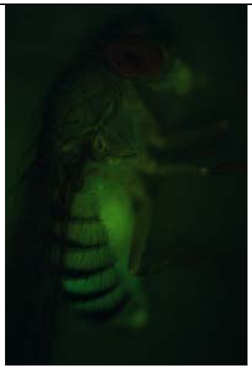	0 ±0	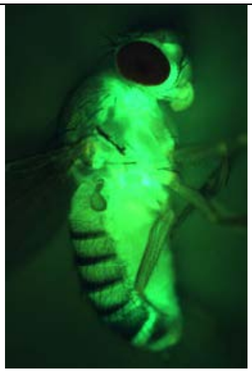	5 ±0
76	CG31937	Dhrs7		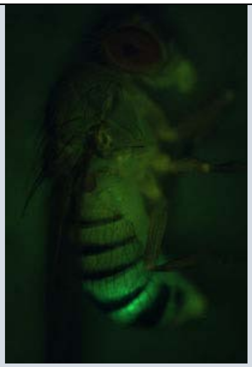	0.2 ±0.1	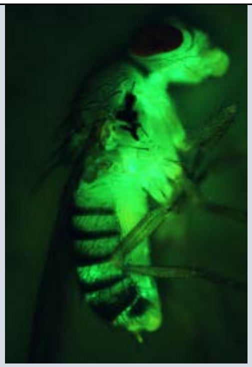	5 ±0
77	CG3454	Hdc		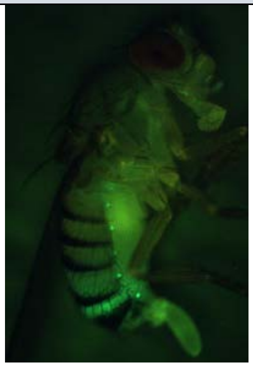	1 ±0.7	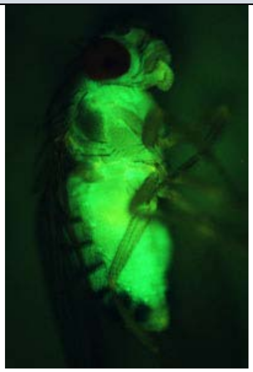	4.9 ±0.13

78	CG10692	Mt2		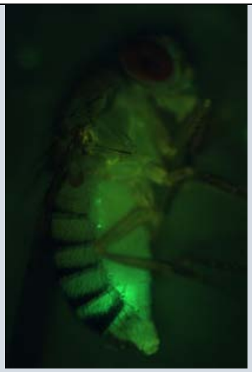	1.2 ±0.7	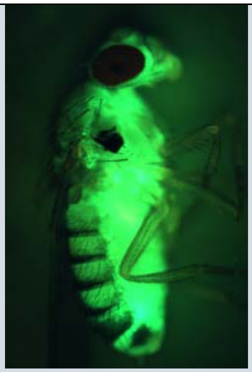	5 ±0
79	CG10388	Hoxa7		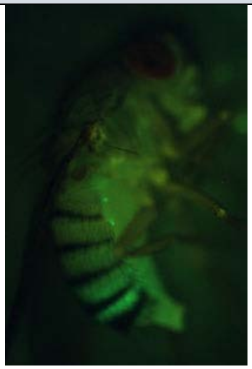	0.4 ±0.2	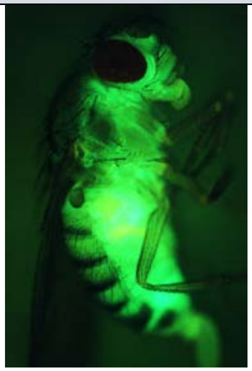	5 ±0
**80**	**CG4583**	**Ern1**		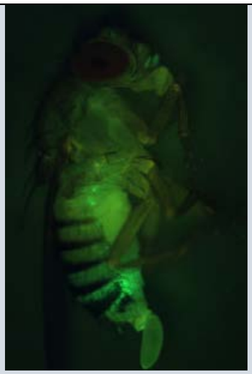	**1 ±0.7**	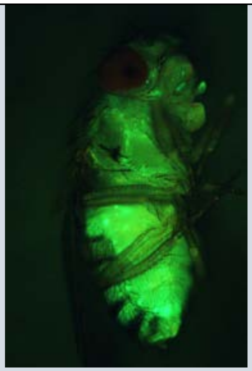	**3 ±0.55**

81	CG5669	Sp4		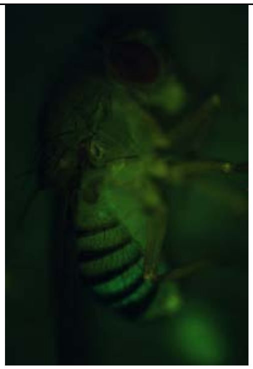	0.3 ±0.1	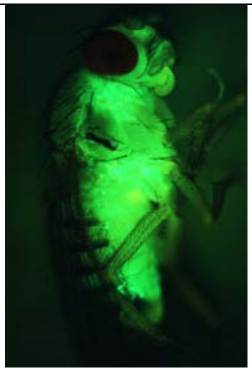	5 ±0
82	CG8804	Ppap2a		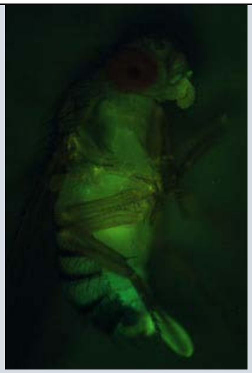	1 ±0.4	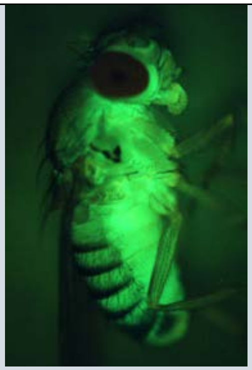	4.7 ±0.24
83	CG11576	2310046K01Rik		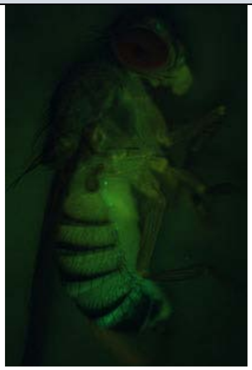	0.5 ±0.3	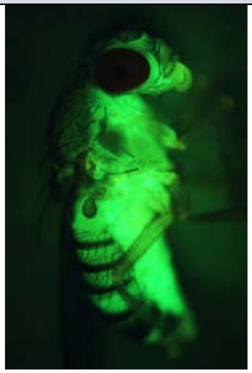	5 ±0

84	CG16868	Cachd1		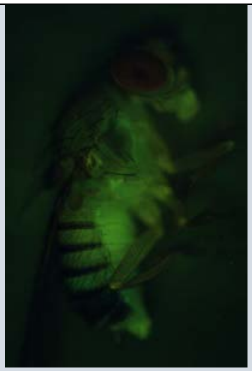	1 ±0.4	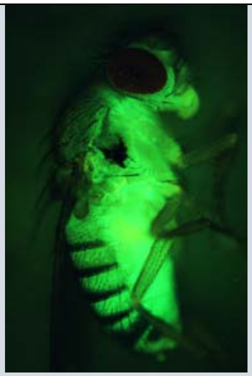	5 ±0
